# Blood pressure and mortality: using offspring blood pressure as an instrument for own blood pressure in the HUNT study

**DOI:** 10.1038/srep12399

**Published:** 2015-07-22

**Authors:** Kaitlin H Wade, David Carslake, Tom Ivar Nilsen, Nicholas J Timpson, George Davey Smith, Pål Romundstad

**Affiliations:** 1 MRC Integrative Epidemiology Unit (IEU) at the University of Bristol, Bristol UK; 2 School of Social and Community Medicine, University of Bristol, Bristol UK; 3 Department of Public Health and General Practice, Norwegian University of Science and Technology, N-7491 Trondheim, Norway

## Abstract

Given that observational associations may be inaccurate, we used offspring blood pressure (BP) to provide alternative estimates of the associations between own BP and mortality. Observational associations between BP and mortality, estimated as hazard ratios (HRs) from Cox regression, were compared to HRs obtained using offspring BP as an instrumental variable (IV) for own BP (N = 32,227 mother-offspring and 27,535 father-offspring pairs). Observationally, there were positive associations between own BP and mortality from all-causes, cardiovascular disease (CVD), coronary heart disease (CHD), stroke and diabetes. Point estimates of the associations between BP and mortality from all-causes, CVD and CHD were amplified in magnitude when using offspring BP as an IV. For example, the HR for all-cause mortality per standard deviation (SD) increase in own systolic BP (SBP) obtained in conventional observational analyses increased from 1.10 (95% CI: 1.09–1.12; *P* < 0.0001) to 1.31 (95% CI: 1.19–1.43; *P* < 0.0001). Additionally, SBP was positively associated with diabetes and cancer mortality (HRs: 2.00; 95% CI: 1.12–3.35; *P* = 0.02 and 1.20; 95% CI: 1.02–1.42; *P* = 0.03, respectively), and diastolic BP (DBP) with stroke mortality (HR: 1.30; 95% CI: 1.02–1.66; *P* = 0.03). Results support positive associations between BP and mortality from all-causes, CVD, and CHD, SBP on cancer mortality, and DBP on stroke mortality.

High blood pressure (BP) is an increasingly important health problem. Studies over the last few decades have confirmed a relationship between high BP and adverse health outcomes, including mortality from all causes^1^,^2^,^3^,^4^,^5^,^6^,^7^,^8^, cancer^9^ and cardiovascular diseases (CVD)^6^, reported within individuals of varying age, socioeconomic status and ethnicity^1^,^2^,^3^,^6^,^8^. A large meta-analysis showed that a difference of 20 mmHg systolic BP (SBP) or 10 mmHg diastolic BP (DBP) was associated with more than a two-fold increase in the risk of death from stroke, ischemic heart disease (IHD) and other vascular causes^6^. Many randomized controlled trials (RCTs) have shown that antihypertensive (BP lowering) medications, including thiazide diuretics, beta blockers, and angiotensin-converting enzyme (ACE) inhibitors, reduce the risk of developing CVD, coronary heart disease (CHD), stroke, and other vascular events and mortality^10^,^11^,^12^,^13^.

Existing literature is inconsistent in explaining the specific pattern of association of BP with all-cause and cause-specific mortality. Many studies have shown a U-shaped relationship, similar to the association observed for body mass index (BMI) and mortality^14^,^15^,^16^,^17^,^18^,^19^,^20^, where individuals with either extremely low or high BP have the highest mortality. Furthermore, there is evidence suggesting an inverse or attenuated association between BP and mortality among the elderly^4^,^14^,^21^,^22^ and a moderate increase in mortality with antihypertensive treatment^23^, specifically for patients on dialysis^24^. Additionally, some antihypertensive medications have been shown to increase type-2 diabetes risk^25^,^26^, which is itself associated with increased BP^27^,^28^. Therefore, it is unclear whether shifting the entire BP distribution downwards would reduce mortality in the whole population.

Most literature on the association between BP and mortality is based on observational analyses that are known to suffer from intractable biases, such as measurement error and confounding^29^, which may attenuate effect sizes or generate spurious results. For example, factors associated with both BP and mortality, such as smoking, BMI, and socioeconomic status, may interfere with the association. Furthermore, conventional observational associations are vulnerable to reverse causation, where disease leading to mortality may also alter BP^30^. Statistical adjustment for confounding factors and methodological removal of deaths occurring shortly after baseline and the first period of follow-up are attempts to overcome such issues inherent to observational data^31^. However, error in BP measurement and unmeasured factors can lead to residual confounding^32^ and biased associations^33^. Consequently, more accurate and reliable estimates of the association between BP and mortality are needed.

A methodological development for the assessment of causality is the application of instrumental variables (IVs)^31^,^34^ within survival analyses. An IV is one that is associated with a risk factor (here, BP) and potentially with an outcome (here, mortality), but only through its association with the risk factor^35^. Additionally, the instrument is not influenced by the confounding factors of the risk factor-outcome association. Given these properties, the variance in a risk factor explained by the instrument provides an unbiased and unconfounded causal assessment of the association between the risk factor and outcome. Importantly for survival analyses, potentially fatal diseases that also affect BP (biasing conventional observational estimates of the effect of BP on mortality) will not be associated with a valid IV. One such application of IV analysis is the use of offspring exposures as instruments for parents’ own exposure (Fig. 1). This method has been applied to investigate the associations between BMI and mortality^31^, height and mortality^36^, and weight and employment disability^34^. The extent to which reverse causation influences the association between BP and mortality was reduced by using offspring exposures compared to own exposures^31^. We therefore used IV analysis to provide a more reliable estimate of the causal associations between BP and mortality, using offspring BP as an instrument for own BP, in data from the Nord-Trøndelag health study (HUNT Study, Norway).

## Results

Data were available for 32,227 mother-offspring pairs (8,907 maternal deaths) and 27,535 father-offspring pairs (9,633 paternal deaths). The residual standard deviation (SD) in SBP/DBP after adjustment for age and HUNT survey (used to scale SBP/DBP) was 17.53 mmHg/10.47 mmHg in women and 16.57 mmHg/10.25 mmHg in men, respectively. Characteristics of included individuals are described in Table 1. Offspring SBP and DBP were positively associated with SBP and DBP of both mothers and fathers (Supplementary Tables S1 and S2, respectively). Parents with higher BP, as well as offspring themselves, had higher BMI, were less likely to have spent ten years in full time education and were more likely to be taking antihypertensive medication. Characteristics of offspring and parents according to maternal and paternal SBP/DBP were similar (Supplementary Tables S3/S4 and S5/S6, respectively).

### Offspring versus own BP

Offspring and own SBP and DBP were positively associated with parental all-cause mortality and mortality from CVD, CHD and stroke (Tables 2 and 3). In each case, associations between own BP and all four primary outcomes were substantially stronger than those obtained with offspring BP.

### IV analysis using offspring BP as an instrument for own BP

For combined parents, the denominators in the IV process (regression coefficients between parental and offspring BP, adjusted for the same variables as the numerator and scaled by sex-specific SD scores) were 0.22 (SE = 0.01) and 0.17 (SE = 0.005) for SBP and DBP, respectively. IV analysis showed positive associations between own BP (using offspring BP as an instrument) and mortality from all causes (HR per SD increase in BP: 1.28; 95% CI: 1.18–1.39; *P* < 0.0001 with SBP and 1.31; 95% CI: 1.19–1.43; *P* < 0.0001 with DBP), CVD (HR per SD increase in BP: 1.38; 95% CI: 1.23–1.54; *P* < 0.0001 with SBP and 1.46; 95% CI: 1.28–1.66; *P* < 0.0001 with DBP), and CHD (HR per SD increase in BP: 1.32; 95% CI: 1.17–1.50; *P* < 0.0001 with SBP and 1.38; 95% CI: 1.19–1.59; *P* < 0.0001 with DBP) (Table 2 and 3 for SBP and DBP, respectively). There was a positive association between own DBP and mortality from stroke (HR per SD increase in DBP: 1.30; 95% CI: 1.02–1.66; *P* = 0.03), the estimate of which was smaller in magnitude with SBP (HR per SD increase in SBP: 1.21; 95% CI: 0.98–1.51; *P* = 0.08); however, the estimate was similar to that obtained in conventional observational analyses.

The HRs from IV regression were substantially greater than those from the corresponding conventional Cox models for mortality from all causes (*P* = 0.0002 with SBP and *P* < 0.0001 with DBP), CVD (*P* = 0.01 with SBP and *P* =0.001 with DBP) and CHD (*P* = 0.02 with SBP and *P* =0.01 with DBP). HRs from IV and conventional Cox regression for stroke were very similar.

In secondary IV analyses, there were positive associations between own SBP and mortality from diabetes, cancer and pancreatic cancer, all HRs of which were greater than those obtained through conventional Cox regression with either offspring or own SBP (Supplementary Tables S7/S8 for SBP/DBP, respectively). The estimates of the associations between own DBP, instrumented by offspring DBP, and mortality from diabetes and cancer were greater (but with wider CIs) compared to conventional observational estimates.

Parent-specific analyses showed similar results (Supplementary Tables S9/S10 and S11/S12 for maternal and paternal SBP/DBP, respectively).

### Sensitivity analyses

Adjusting for BMI made little difference in most conventional Cox regressions (Supplementary Tables S13/S14 for SBP/DBP, respectively). However, the IV-estimated HRs for mortality from CHD increased slightly. In IV analyses, adjusting for BMI gave similar HRs for mortality from all causes and CVD.

Excluding individuals taking antihypertensive medication produced similar results (Supplementary Tables S15/S16 for SBP/DBP, respectively). However, the IV-estimated HRs for CVD mortality attenuated towards the null. IV-estimated HRs for all other primary causes of mortality were consistent with main results.

In secondary IV analyses, the HRs for diabetes mortality attenuated towards the null when adjusting for BMI (HR per SD increase in BP: 1.79; 95% CI: 0.95–3.36; *P* = 0.07 with SBP and 1.72; 95% CI: 0.81–3.63; *P* = 0.16 with DBP). After excluding individuals taking antihypertensive medication, the IV-estimated HRs for mortality from diabetes, cancer and pancreatic cancer with own SBP and the IV-estimated HRs for mortality from diabetes, respiratory diseases and colorectal cancer with DBP increased, although CIs became wider.

### Linearity of mortality using offspring and own BP

**Figure 2:**
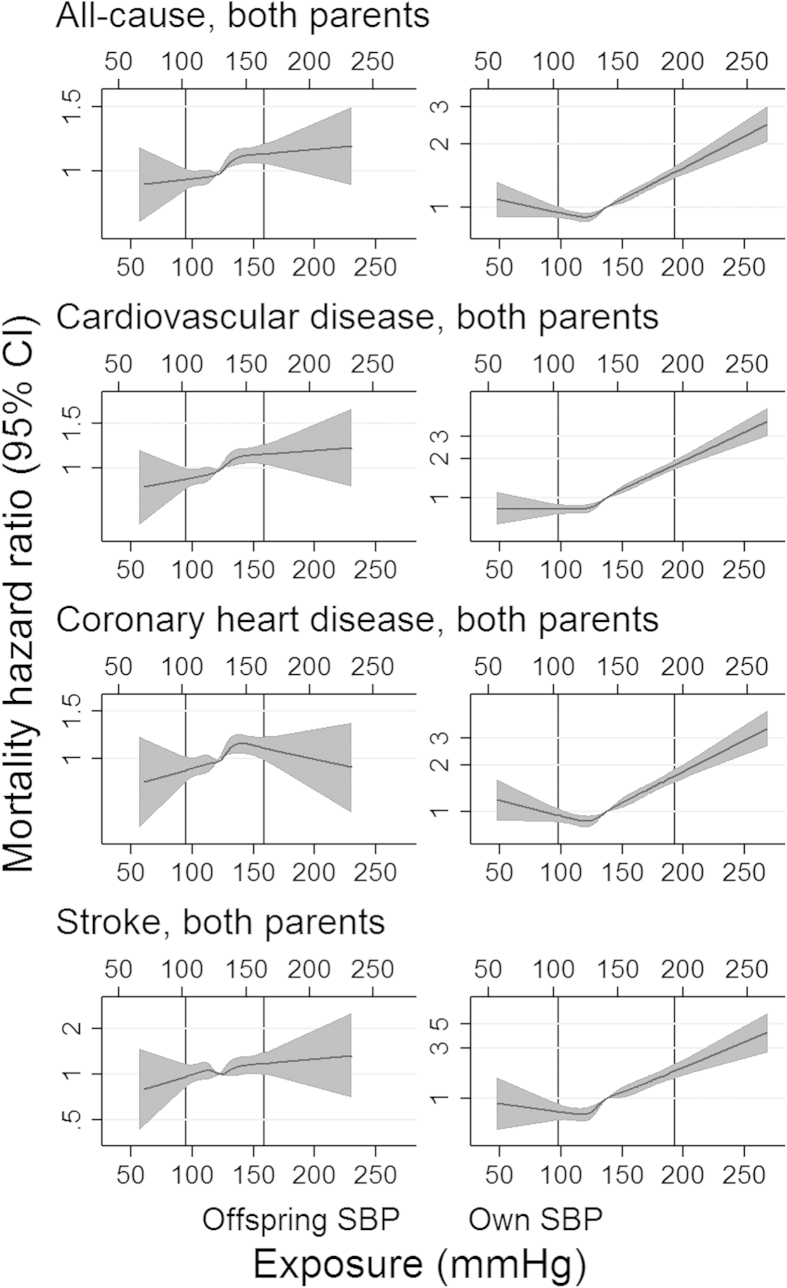
Fitted HRs (median SBP as a reference) from cubic spline models for selected mortality causes. Shaded areas represent 95% CI and vertical lines represent the 1^st^ and 99^th^ percentiles of systolic blood pressure (SBP). Although similar, the upper X-axis applies to male SBP and the lower X-axis to female SBP.

**Figure 3:**
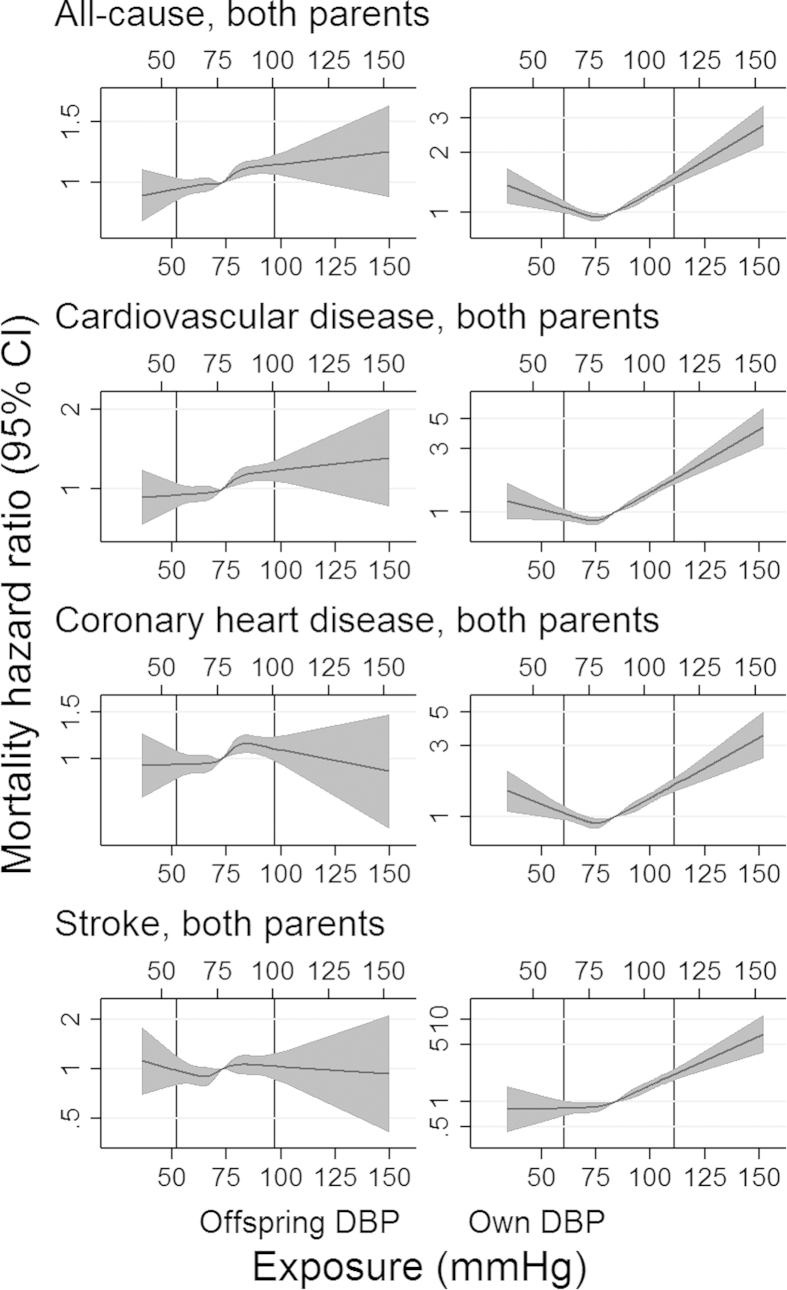
Fitted HRs (median DBP as a reference) from cubic spline models for selected mortality causes. Shaded areas represent 95% CI and vertical lines represent the 1st and 99th percentiles of diastolic blood pressure (DBP). Although similar, the upper X-axis applies to male DBP and the lower X-axis to female DBP.

Due to differences in the SD of BP between men and women, conversion of units back from SD to mmHg led to slightly different values according to the sex of the individual. Therefore, the exposure scales for men and women in the cubic spline plots, although similar, are represented on the upper and lower x-axes, respectively. Assessment of linearity between own BP and most causes of mortality showed a U-shaped association. However, this pattern was not observed with offspring BP (Figs 2 and 3 for associations of SBP and DBP with all-cause mortality and mortality from CVD, CHD and stroke; Supplementary Figures S3-S8 for all mortality outcomes).

## Discussion

This is the first large prospective study to use offspring BP as an instrument for own BP to obtain more reliable estimates of causal associations between BP and mortality, without the limitations inherent in conventional observational analyses. Results provided evidence to support positive causal associations between own BP and mortality from all causes, CVD and CHD; and own DBP and stroke mortality; and own SBP and mortality from cancer, pancreatic cancer and diabetes. IV-estimated HRs were larger than those obtained from conventional Cox regression, highlighting the potential confounding within conventional observational associations. The positive association between own SBP and mortality from diabetes attenuated with adjustment for BMI, suggesting either mediation through BMI or an independent association between BMI and mortality from diabetes. The estimate of the positive association between own SBP and mortality from pancreatic cancer was found using IV but not in conventional observational analyses, suggesting that observational analyses may underestimate the causal relationship. However, the potential role of chance in this and other relatively weak associations such as those observed between own SBP and mortality from the various other cancers cannot be ruled out. When excluding individuals taking antihypertensive medication, HRs for a majority of mortality causes remained consistent with main analyses; however, the HR for CVD mortality attenuated towards the null, highlighting the potential overestimation of estimates owing to the use of antihypertensives.

Our main findings are consistent with previous studies showing an increased risk of all-cause mortality and mortality from CVD and CHD with increasing BP^1^,^2^,^5^,^6^,^7^,^8^,^10^,^11^,^12^. Our results are also in line with many studies showing a positive association between hypertension and diabetes, which tend to occur simultaneously^27^,^28^, where the direction of causality is unclear. Our results suggest a causal association with increasing BP and an increased risk of diabetes mortality; however, there are many factors that may confound the association^37^,^38^. Upon adjustment for BMI in IV analyses, the HRs for diabetes mortality decreased, suggesting that parents’ BMI may be associated both with their own risk of diabetic mortality and with their offspring’s BP (probably via the offspring’s BMI)^31^. Our BMI-adjusted results are also consistent with the Prospective Studies Collaboration, which showed that individuals with a BMI above 22.5–25 kg/m^2^ have an increased risk of diabetic, renal and vascular mortality^39^. Additionally, evidence on the effect on diabetes of strategies to reduce BP is inconclusive^25^,^26^,^27^,^28^. Although CIs widened, excluding individuals known to be taking antihypertensives in our analyses increased the IV HRs for mortality from diabetes.

Similar to the conventional observational associations found between own BP and mortality, previous studies have reported a U-shaped association between mortality and BP^14^,^15^,^16^,^17^,^18^,^19^,^20^; however, this was not supported when using offspring BP. As many of these studies used populations with a previous illness^14^,^15^,^16^,^17^,^18^,^19^,^20^, the nonlinear associations may be explained by the presence of such previous illnesses, which may potentially lower BP and increase risk of mortality, producing a reverse association. In fact, larger studies assessing the association between BP and mortality, which did adjust for baseline traits, provided more evidence for a linear relationship^14^,^15^,^16^,^17^,^18^,^19^,^20^. Our current finding supports this linear association, suggesting that the U-shaped curve found in many observational studies may be due to reverse causation or confounding, underestimating the positive effect of elevated BP on mortality, but falsely overestimating the effect of low BP on mortality.

As previously discussed^31^, the avoidance of reverse causation depends on the IV satisfying some important assumptions. Of these, we showed that the instrument (here, offspring BP) was associated with the exposure (here, own BP). Secondly, the extent to which reverse causation influenced the association between BP and mortality was reduced by using offspring BP compared to own BP^31^. It is unlikely that fathers’ illness would directly affect offspring BP^31^; however, mothers’ illness (especially during pregnancy) may later influence fetal development, and thus BP. Therefore, confounding may still be present in such associations, specifically in mother-offspring pairs, if intrauterine exposures are associated with offspring BP (for example smoking during pregnancy has been shown to be associated with offspring BP during later life^40^,^41^). Such exposures are likely to lead to differences in mortality estimates between mother- and father-offspring pairs, which we do not observe. Additionally, the earliest available HUNT survey was used to maximise the period that parents were at risk and minimize the potential influence of illness-induced alterations to both offspring and own BP. Therefore, it is unlikely that the instrument was affected by the outcome.

Thirdly, although we could not test whether the instrument was independent of any residual (or unmeasured) confounding factors, or if there were any other pathways from the instrument to the outcome directly (regardless of the exposure), reassuringly, the adjustment for measured potential confounders made little difference to results. Whilst the IV methodology used here cannot be considered gold standard for assessing causality, results add to existing literature within this context. Shared environmental confounding, specifically between the instrument (here, offspring BP) and the outcome (mortality), may still be a potentially confounding factor when using offspring measures as instruments for own measures. However, the inherent correlation between offspring and parental BP measures and likely differences in transgenerational environment would be more likely to break down confounding structures and reduce bias in these analyses, and as such be an improvement to observational measures alone. Finally, although it is often difficult to refute the existence of independent pathways from the instrument to the outcome, it is unlikely that offspring BP could directly affect parental mortality.

Although we adjusted participants’ BP for the treatment effect of antihypertensives, and performed a sensitivity analysis excluding individuals taking such medication, there may have been antihypertensive use at baseline or measurement error (as antihypertensive use was self-reported).

Contrary to previous reports of U-shaped associations between BP and mortality, this pattern was observed for own BP, but not for offspring BP, suggesting that the causal association between BP and mortality is approximately log-linear (due to the assumption of log-linearity in the IV analyses). However, as some associations between offspring BP and cause-specific mortality (CHD, stroke, breast cancer and prostate cancer, for example) showed a nonlinear pattern, interpretation and generalization of the IV approach may differ.

Despite these limitations, our current study had sufficient power to make reliable estimates of the likely causal association between BP and mortality, while adjusting for appropriate confounding factors and using family data to compare IV and conventional observational estimates. Although the IV approach produced considerably less precise estimates (wider CIs than in conventional observational analyses), using offspring BP as an instrument for own BP provided a more reliable estimate of the causal relationship between BP and mortality, without limitations inherent in conventional observational studies. Results are likely to be generalisable to other European populations, but may be less relevant for less developed or for ethnically diverse populations.

Results support the potentially causal role of elevated BP on all-cause mortality and mortality from CVD and CHD, elevated DBP and stroke mortality, and elevated SBP on mortality from diabetes. The positive association between elevated SBP and diabetes mortality attenuated upon adjustment for BMI, either suggesting that the relationship is, in part, mediated through BMI or there is an independent association between BMI and diabetes mortality. Therefore, clinical interventions to reduce diabetes mortality should potentially focus primarily on BMI. Importantly, using IV methodology, the previously reported U-shaped association between BP and mortality was not observed, suggesting that an increased mortality with lower levels of BP may be a result of reverse causation or confounding. As IV-estimated HRs were larger than those obtained from conventional Cox regression, taken together, these results highlight the adverse causal effect of high BP on later mortality risk.

## Methods

The HUNT Study is a large-scale prospective study of family and individual data on approximately 125,000 individuals living in the rural Nord-Trøndelag County in central Norway. The data can be linked to national and regional health registries and contain additional information from The Population Census Register, National Population Registry and Family Register, which are provided by Statistics Norway to create a genealogical database and emigration status^42^. The Cause of Death Registry provided by Statistics Norway was used to identify all deaths among participants between the beginning of HUNT1 (1^st^ January 1984) and 31^st^ December 2009, classified by cause according to their international classification of diseases (ICD) code (Supplementary Table S17).

Methods and background of the HUNT study have been described previously^42^,^43^. The study consists of three consecutive surveys at which residents of aged 20 years and older were invited to participate. An adolescent part (Young-HUNT Study) included people aged 13–19 years old was also conducted. In the first HUNT survey (HUNT1; 1984–1986), 74,599 individuals were successfully recruited (88.1% response rate). At the second survey (HUNT2; 1995–1997) questionnaire-based information and blood samples were obtained from approximately 65,000 individuals (69.5% response rate). In HUNT3 (2008), data were collected through questionnaires, interviews, clinical examinations, and blood and urine samples from 48,289 participants (52% response rate). Additionally, Young-HUNT^44^ included data from self-reported questionnaires, interviews, and clinical measurements: Young-HUNT1 (1995–1997; n = 9,141 (90% response rate)), Young-HUNT2 (2000–2001; n = 2,400 (77% response rate)), and Young-HUNT3 (2006–2008; n = 8,677 (87% response rate)).

Initially, HUNT or Young-HUNT participants who had at least one participating parent were extracted (66,262 offspring, with 62,414 participating mothers and 53,145 participating fathers). Participants at every HUNT and Young-HUNT survey attended a physical examination, where SBP and DBP were measured. If individuals participated in multiple surveys, data from the earliest HUNT survey were used to maximise the period that parents were at risk and minimize the potential influence of illness-induced alteration of BP. Young-HUNT data were used for offspring only when no HUNT data were available, to increase sample size and minimize variation in BP due to different stages of adolescence. Data were restricted to one randomly chosen offspring per parent and, where possible, the same offspring was chosen for both parents. Data were available for 32,227 mother-offspring pairs (15,941/16,286 daughters/sons) and 27,535 father-offspring pairs (13,623/13,912 daughters/sons) (Supplementary Figure S1).

### Statistical Analysis

The analyses were performed with primary focus on all-cause mortality and mortality specifically from CVD, CHD and stroke. Secondary analyses examined mortality from diabetes, various cancers, respiratory diseases and external causes (presented in the Supplementary Material). Each measure of offspring and own BP was assigned to quintiles among participants of the same sex and similar age, in the same HUNT survey. Within these quintiles, we summarized offspring and own health-related characteristics and assessed the corresponding association with BP using linear and logistic regression appropriately. As recommended by Tobin *et al.*^45^, we adjusted for the treatment effect of antihypertensives by adding 10 mmHg or 5 mmHg onto the SBP or DBP measurements, respectively, of individuals who self-reported taking BP-lowering medication before all analyses. Individuals with missing data on antihypertensive use were excluded (Supplementary Figure S1). As Young-HUNT participants were not asked about medication, they were assumed not to be using antihypertensives due to their young age and were not excluded.

Before all analyses, offspring and own BP values were converted to sex-specific Z-scores, adjusted for age (cubic spline with five knots at the 5^th^, 27.5^th^, 50^th^, 72.5^th^, and 95^th^ percentiles) interacting with HUNT survey (a categorical variable based on HUNT/Young-HUNT) We used the Cox proportional hazard model with own age as the time axis to estimate hazard ratios (HRs) per standard deviation (SD) increase in offspring and own HUNT survey- and age-adjusted BP. To account for clustering by offspring identity we used robust standard errors (SEs). All models were adjusted for own sex and date of birth (DOB; to account for secular trends in mortality). Fully-adjusted models additionally included own smoking status, alcohol consumption, education, exercise, own and spouse’s employment, and offspring’s smoking status. As unadjusted and fully-adjusted estimates were similar, only fully-adjusted models are presented (unadjusted analyses available on request). Heterogeneity between maternal and paternal HRs was tested by adding an interaction between parental sex and the exposure into the model and examining the p-value of the resulting coefficient. As there was little evidence for heterogeneity (Supplementary Tables S18/S19 for SBP/DBP, respectively), mothers and fathers were analysed together. Parent-specific analyses, not adjusted for parental sex and not requiring robust SEs, were conducted as a supplement.

To avoid reverse causation, IV analysis was used to estimate HRs per SD of own BP, using offspring BP as an IV for own BP (Supplementary Figure S2). Firstly, own BP was regressed on offspring BP, with adjustment for all covariables (own and parental smoking status, age, DOB, alcohol consumption, education, own and spouse’s employment exercise and sex of parent). The causal HRs per SD of own BP were then estimated by exponentiating the ratio between the natural logarithm of the corresponding HR per SD increase in offspring BP, obtained through conventional Cox regression with the same adjustment, and the adjusted regression coefficient for own BP against offspring BP. Confidence intervals (CIs) were calculated using Taylor series expansions^46^. The HRs obtained through IV analyses were compared with conventional observational HRs per SD of own BP using 1,000 bootstrap samples. In a sensitivity analysis, own BMI was added into fully-adjusted models. In a second sensitivity analysis, we excluded all individuals who self-reported taking antihypertensives (See Supplementary Methods for IV equation).

Linearity of associations was tested by plotting fully-adjusted cubic spline models for both offspring and own BP to show the pattern of association between BP and mortality. Observations were censored to restrict them to the period when death would have been recorded and used in the analysis of own BP and offspring BP. Analyses were performed using STATA versions 12 and 13 (Stat Corp, Texas).

## Additional Information

**How to cite this article**: Wade, K. H. *et al.* Blood pressure and mortality: using offspring blood pressure as an instrument for own blood pressure in the HUNT study. *Sci. Rep.* **5**, 12399; doi: 10.1038/srep12399 (2015).

## Supplementary Material

Supplementary Information

## Figures and Tables

**Figure 1:**
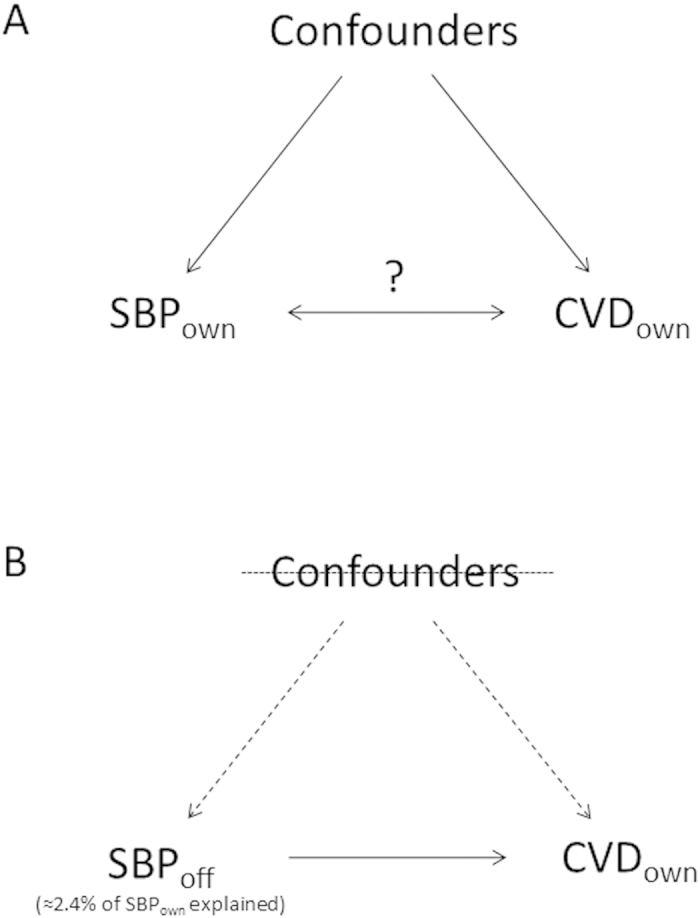
Diagram representing the underlying IV methodology employed in analyses using the example of assessing the association between SBP and CVD mortality. **A**) In observational epidemiology, the association between the exposure (here, own SBP (SBP_own_)) and an outcome of interest (here, own mortality from CVD (CVD_own_)) may be distorted due to confounding, bias and reverse causation. **B**) Using offspring SBP (SBP_off_) as an instrument for SBP_own_ (where SBP_off_ explains ~2.4% of the variance in SBP_own_) reduces the possibility of such limitations. Firstly, unlike observational analyses, the portion of variance in SBP_own_ explained by SBP_off_ will not be directly affected by CVD_own_; therefore, reducing bias due to reverse causation. Secondly, the effect of potential confounding variables is reduced because they are less associated with SBP_off_ than they are with SBP_own_. Any remaining confounding due to the association between confounders and SBP_off_ can be further reduced by adjustment for shared and measured environmental confounders (dotted lines).

**Table 1:** Descriptive characteristics of the study population.

Subject, Measurement	N	Mean (SD) or proportion
Offspring		
Age (years)	63,807	29.54 (10.94)
Sex (% male)	66,262	51.15
BMI (kg/m^2^)	63,578	24.14 (4.02)
SBP (mmHg)	63,807	126.25 (14.53)
DBP (mmHg)	63,807	75.02 (11.61)
Proportion ever smoked (%)	57,940	41.92
Proportion drinking ≥5 times fortnightly (%)	43,823	4.11
Proportion educated ≥10 years (%)	37,420	72.07
Proportion physically active (%)	38,167	90.68
Proportion taking antihypertensives (%)	50,418	3.09
Mothers		
BMI (kg/m^2^)	61,163	25.53 (4.56)
SBP (mmHg)	61,526	135.81 (25.03)
DBP (mmHg)	61,526	82.60 (12.25)
Age at child’s birth (years)	61,526	49.07 (16.17)
Proportion ever smoked (%)	52,655	45.52
Proportion drinking ≥5 times fortnightly (%)	51,542	2.77
Proportion educated ≥ 10 years (%)	50,987	39.07
Proportion physically active (%)	43,352	83.95
Proportion taking antihypertensives (%)	61,491	15.35
Fathers		
BMI (kg/m^2^)	52,226	25.58 (3.23)
SBP (mmHg)	52,452	140.04 (20.35)
DBP (mmHg)	52,452	85.87 (11.28)
Age at child’s birth (years)	52,452	50.33 (15.95)
Proportion ever smoked (%)	45,111	64.11
Proportion drinking ≥5 times fortnightly (%)	44,109	8.20
Proportion educated ≥10 years (%)	42,990	45.98
Proportion physically active (%)	36,726	84.46
Proportion taking antihypertensives (%)	52,413	10.83

BMI: body-mass index, CI: confidence interval, DBP: diastolic blood pressure, SBP: systolic blood pressure, SD: standard deviation.

**Table 2:** Adjusted HRs per SD of offspring SBP for combined parents and HRs for own SBP estimated by conventional observational and IV analyses.

Cause of Death	Deaths	Offspring SBP, fully adjusted*			Own SBP, fully adjusted*			IV, fully adjusted*				P-value for comparison with own SBP
		HR	95% CI	P-value	HR	95% CI	P-value	HR	95% CI	P-value		
All cause	18,540	1.05	(1.04–1.07)	<0.0001	1.11	(1.09–1.12)	<0.0001	1.28	(1.18–1.39)	<0.0001		0.0002
Cardiovascular Disease	8,772	1.07	(1.05–1.10)	<0.0001	1.19	(1.17–1.21)	<0.0001	1.38	(1.23–1.54)	<0.0001		0.01
Coronary Heart Disease	6,571	1.06	(1.03–1.09)	<0.0001	1.15	(1.13–1.17)	<0.0001	1.32	(1.17–1.50)	<0.0001		0.02
Stroke	2,268	1.04	(0.99–1.09)	0.08	1.22	(1.18–1.26)	<0.0001	1.21	(0.98–1.51)	0.08		0.95

CI: confidence interval, HR: hazard ratio, IV: instrumental variable, SBP: systolic blood pressure, SD: standard deviation.

^*^adjusted for age, sex and HUNT survey of offspring/parent from whom SBP was measured, offspring and parental smoking status, age, DOB, alcohol consumption, education, own and spouse’s employment, exercise and sex of parent.

**Table 3:** Adjusted HRs per SD of offspring DBP for combined parents and HRs for own DBP estimated by conventional observational and IV analyses.

Cause of Death	Deaths	Offspring DBP, fully adjusted*			Own DBP, fully adjusted*			IV, fully adjusted*			P-value for comparison with own DBP
		HR	95% CI	P-value	HR	95% CI	P-value	HR	95% CI	P-value	
All cause	18,540	1.05	(1.03–1.06)	<0.0001	1.10	(1.09–1.12)	<0.0001	1.31	(1.19–1.43)	<0.0001	0.0001
Cardiovascular Disease	8,772	1.07	(1.04–1.09)	<0.0001	1.19	(1.16–1.21)	<0.0001	1.46	(1.28–1.66)	<0.0001	0.001
Coronary Heart Disease	6,571	1.06	(1.03–1.08)	<0.0001	1.14	(1.11–1.16)	<0.0001	1.38	(1.19–1.59)	<0.0001	0.01
Stroke	2,268	1.05	(1.00–1.09)	0.03	1.26	(1.21–1.31)	<0.0001	1.30	(1.02–1.66)	0.03	0.81

CI: confidence interval, DBP: diastolic blood pressure, HR: hazard ratio, IV: instrumental variable, SD: standard deviation.

^*^adjusted for age, sex and HUNT survey of offspring/parent from whom DBP was measured, offspring and parental smoking status, age, DOB, alcohol consumption, education, own and spouse’s employment, exercise and sex of parent.
