# Supplementary material for: Blood pressure and mortality: using offspring blood pressure as an instrument for own blood pressure in the HUNT study
Source: Sci Rep. 2015 Jul 22;5:12399. doi: 10.1038/srep12399 (PMC4510525; doi:10.1038/srep12399)
Supplement: Supplementary Information [file srep12399-s1.pdf]

Supplementary material for the manuscript entitled “Blood pressure and mortality: using offspring blood pressure as an instrument for own blood pressure in the HUNT study”

Kaitlin H Wade, David Carslake, Tom Ivar Nilsen, Nicholas J Timpson, George Davey Smith, Pål Romundstad

SUPPLEMENTARY METHODS

Instrumental Variable Methodology

The causal association between own blood pressure (BP) and mortality was estimated by using offspring BP as an instrument for own BP to avoid the issues of reverse causation and confounding. The equation below illustrates this methodology, where Z represents the instrument (here, offspring BP), X represents the exposure (here, own BP), and Y represents the outcome (here, all-cause or cause-specific mortality).

$$HR_{XY} = exp\left(\frac{Ln(HR_{ZY})}{\beta_{ZX}}\right)$$

Firstly, own BP was regressed on offspring BP (giving the coefficient β_{ZX}), with adjustment for all covariables (age and sex of offspring, HUNT survey, own and parental smoking status, age, date of birth, alcohol consumption, education, own and spouse's employment exercise and sex of parent) and scaled by sex-specific standard deviation (SD) scores. The causal hazard ratios (HRs) (HR_{XY}) per SD of own BP were then estimated by exponentiating the ratio between the natural logarithm of the corresponding HR per SD increase in offspring BP (HR_{ZY}), obtained through

conventional Cox regression with the same adjustment, and the adjusted regression coefficient for own BP against offspring BP (β_{ZX}). Confidence intervals (CIs) were calculated using Taylor series expansions[1]. The HRs obtained through instrumental variable (IV) analyses were compared with conventional observational HRs per SD of own BP using 1,000 bootstrap samples.

SUPPLEMENTARY TABLES

Supplementary Table S1. Properties of offspring and parents according to quintiles of offspring SBP

Subject, Measurement	Quintile of offspring SBP					Linear or logistic regression per SD*		
	1st	2nd	3rd	4th	5th	Regression Coefficient or OR†	95% CI	N
**Offspring**								
Mean BMI (kg/m^2)	22.9	23.5	23.9	24.5	25.5	1.27	(1.21–1.32)	34,942
Mean SBP (mmHg)	110.2	119.3	125.5	132.3	145.4	17.31	(17.21–17.40)	35,072
Mean DBP (mmHg)	67.3	71.5	74.1	77.2	82.1	7.39	(7.25–7.54)	35,072
Proportion ever smoked (%)	40.5	38.6	38.1	38.8	37.7	0.96	(0.93–0.99)	32,117
Proportion drinking ≥ 5 times fortnightly (%)	3.5	4.0	4.4	4.3	4.1	1.07	(0.99–1.17)	22,457
Proportion educated ≥ 10 years (%)	76.9	74.3	72.5	72.6	70.6	0.91	(0.87–0.94)	18,621
Proportion physically active (%)	90.8	91.1	91.3	90.0	91.2	1.01	(0.95–1.07)	19,743
Proportion taking antihypertensives (%)	0.9	1.6	2.3	4.0	7.6	2.32	(2.16–2.50)	25,833
**Mothers**								

Mean BMI (kg/m^2)	25.0	25.1	25.1	25.4	25.7	0.35	(0.28–0.41)	32,558
Mean SBP (mmHg)	131.6	133.8	135.3	137.7	140.7	4.45	(4.07–4.82)	32,767
Mean DBP (mmHg)	79.9	80.8	81.5	82.4	83.8	1.87	(1.70–2.05)	32,767
Mean age at child's birth (years)	27.3	27.4	27.3	27.4	27.4	0.04	(-0.04–0.12)	32,948
Proportion ever smoked (%)	47.1	46.8	49.4	46.6	47.7	1.00	(0.97–1.04)	28,145
Proportion drinking ≥ 5 times fortnightly (%)	2.8	2.8	3.0	3.0	2.3	0.94	(0.86–1.04)	27,550
Proportion educated ≥ 10 years (%)	45.9	44.1	44.5	42.9	41.2	0.91	(0.88–0.94)	26,971
Proportion physically active (%)	86.0	85.0	84.7	84.6	85.3	0.98	(0.94–1.03)	23,299
Proportion taking antihypertensives (%)	10.6	12.1	13.8	15.9	18.7	1.38	(1.33–1.44)	32,753
**Fathers**								
Mean BMI (kg/m^2)	25.3	25.4	25.5	25.6	25.7	0.20	(0.15–0.25)	27,807
Mean SBP (mmHg)	136.6	138.6	139.6	141.7	144.4	3.83	(3.49–4.16)	27,920
Mean DBP (mmHg)	83.6	84.6	85.0	86.0	86.9	1.62	(1.44–1.80)	27,920
Mean age at child's birth (years)	30.4	30.6	30.3	30.5	30.6	0.05	(-0.05–0.15)	28,175
Proportion ever smoked (%)	64.0	63.7	63.3	63.2	63.2	0.99	(0.96–1.03)	24,007
Proportion drinking ≥ 5 times fortnightly (%)	8.6	8.3	8.3	8.3	6.7	0.92	(0.86–0.98)	23,467

Proportion educated ≥ 10 years (%)	52.0	50.5	50.9	48.3	46.9	0.90	(0.87–0.94)	22,738
Proportion physically active (%)	85.5	84.6	85.8	84.2	84.6	0.98	(0.93–1.03)	19,663
Proportion taking antihypertensives (%)	7.3	8.7	10.0	10.8	13.2	1.36	(1.29–1.43)	27,903

BMI: body-mass index, CI: confidence interval, DBP: diastolic blood pressure, OR: odds ratio, SBP: systolic blood pressure, SD: standard deviation

**adjusted for age, sex and HUNT survey*

†Continuous variables are represented as regression coefficients and proportions are represented as ORs per SD increase in offspring SBP

Supplementary Table S2. Properties of offspring and parents according to quintiles of offspring DBP

Subject, Measurement	Quintile of offspring DBP					Linear or logistic regression per SD*		
	1st	2nd	3rd	4th	5th	Regression Coefficient or OR†	95% CI	N
**Offspring**								
Mean BMI (kg/m²)	23.2	23.7	23.8	24.3	25.2	0.80	(0.75–0.85)	34,942
Mean SBP (mmHg)	116.8	121.9	125.2	129.2	137.7	8.75	(8.61–8.90)	35,072
Mean DBP (mmHg)	62.5	69.9	74.3	79.0	87.7	10.47	(10.39–10.56)	35,072
Proportion ever smoked (%)	40.3	39.6	38.8	37.7	37.3	0.94	(0.92–0.96)	32,117
Proportion drinking ≥ 5 times fortnightly (%)	4.0	3.4	3.9	4.2	4.9	1.13	(1.05–1.21)	22,457
Proportion educated ≥ 10 years (%)	75.5	73.3	74.1	73.3	70.8	0.96	(0.93–1.00)	18,621
Proportion physically active (%)	90.9	91.1	90.6	91.3	90.5	0.98	(0.93–1.03)	19,743
Proportion taking antihypertensives (%)	0.9	1.6	1.9	3.5	8.6	2.52	(2.36–2.70)	25,833
**Mothers**								
Mean BMI (kg/m²)	25.1	25.2	25.1	25.3	25.6	0.28	(0.22–0.33)	32,558

Mean SBP (mmHg)	131.9	133.0	134.1	135.2	137.8	2.62	(2.32–2.92)	32,767
Mean DBP (mmHg)	80.1	81.4	82.2	83.2	85.1	2.03	(1.87–2.19)	32,767
Mean age at child's birth (years)	27.4	27.4	27.3	27.4	27.4	0.07	(0.00–0.14)	32,948
Proportion ever smoked (%)	47.7	47.3	47.6	47.4	47.7	0.97	(0.95–1.00)	28,145
Proportion drinking ≥ 5 times fortnightly (%)	2.9	2.7	3.4	2.5	2.5	0.96	(0.89–1.04)	27,550
Proportion educated ≥ 10 years (%)	44.8	44.6	43.4	43.5	42.5	0.94	(0.91–0.97)	26,971
Proportion physically active (%)	85.1	85.6	85.1	85.2	84.6	1.00	(0.96–1.04)	23,299
Proportion taking antihypertensives (%)	11.0	13.2	13.4	15.3	18.1	1.29	(1.24–1.33)	32,753
**Fathers**								
Mean BMI (kg/m^2)	25.3	25.4	25.5	25.6	25.6	0.16	(0.12–0.20)	27,807
Mean SBP (mmHg)	136.7	138.2	138.7	140.1	142.0	2.32	(2.06–2.59)	27,920
Mean DBP (mmHg)	83.4	84.8	85.7	86.6	88.1	1.83	(1.68–1.99)	27,920
Mean age at child's birth (years)	30.5	30.5	30.4	30.5	30.6	0.08	(0.00–0.16)	28,175
Proportion ever smoked (%)	63.4	62.9	64.7	63.4	63.0	0.98	(0.96–1.01)	24,007
Proportion drinking ≥ 5 times fortnightly (%)	8.1	8.4	8.5	7.8	7.3	0.95	(0.90–1.00)	23,467
Proportion educated ≥ 10 years (%)	50.7	50.4	49.0	50.9	47.7	0.94	(0.91–0.97)	22,738

Proportion physically active (%)	85.4	84.6	85.5	84.8	84.5	0.98	(0.94–1.03)	19,663
Proportion taking antihypertensives (%)	7.4	8.7	10.3	10.8	12.8	1.32	(1.26–1.38)	27,903

BMI: body-mass index, CI: confidence interval, DBP: diastolic blood pressure, OR: odds ratio, SBP: systolic blood pressure, SD: standard deviation

*adjusted for age, sex and HUNT survey

†*Continuous variables are represented as regression coefficients and proportions are represented as ORs per SD increase in offspring DBP*

Supplementary Table S3. Properties of offspring and parents according to quintiles of mother's SBP

Subject, Measurement	Quintile of mother's SBP					Linear or logistic regression per SD*		
	1st	2nd	3rd	4th	5th	Regression Coefficient or OR†	95% CI	N
**Offspring**								
Mean BMI (kg/m²)	23.7	23.8	24.0	24.2	24.4	0.19	(0.15–0.23)	32,107
Mean SBP (mmHg)	123.1	124.6	125.9	127.4	129.2	1.96	(1.81–2.11)	32,227
Mean DBP (mmHg)	72.2	73.2	74.0	74.9	76.0	1.11	(0.99–1.23)	32,227
Proportion ever smoked (%)	37.6	37.2	38.4	38.6	39.4	1.01	(0.99–1.03)	29,572
Proportion drinking ≥ 5 times fortnightly (%)	4.7	4.0	3.6	3.5	3.7	0.93	(0.88–0.99)	20,690
Proportion educated ≥10 years (%)	77.0	75.5	74.2	74.9	72.8	0.93	(0.91–0.96)	17,078
Proportion physically active (%)	90.9	91.1	90.0	91.2	91.8	1.05	(1.00–1.09)	18,255
Proportion taking antihypertensives (%)	2.2	2.9	2.7	3.4	3.9	1.17	(1.10–1.24)	23,738
**Mothers**								
Mean BMI (kg/m²)	23.9	24.7	25.3	25.8	26.8	0.89	(0.85–0.94)	32,018

Mean SBP (mmHg)	113.2	125.5	134.9	144.9	165.5	18.41	(18.22–18.59)	32,227
Mean DBP (mmHg)	72.7	78.1	81.6	85.4	92.2	6.31	(6.21–6.42)	32,227
Mean age at child's birth (years)	27.5	27.3	27.3	27.4	27.3	-0.04	(-0.10–0.02)	32,227
Proportion ever smoked (%)	51.8	48.1	45.9	44.9	44.1	0.90	(0.88–0.92)	27,695
Proportion drinking ≥ 5 times fortnightly (%)	3.2	2.9	2.8	2.7	2.3	0.91	(0.84–0.97)	27,107
Proportion educated ≥10 years (%)	49.6	46.6	43.0	40.7	38.1	0.88	(0.86–0.90)	26,534
Proportion physically active (%)	86.7	86.3	84.6	84.5	83.2	0.92	(0.89–0.95)	22,955
Proportion taking antihypertensives (%)	5.9	10.4	13.1	17.9	25.4	1.70	(1.65–1.75)	32,227
**Fathers**								
Mean BMI (kg/m^2)	25.3	25.4	25.5	25.6	25.6	0.09	(0.05–0.12)	25,008
Mean SBP (mmHg)	137.6	138.6	139.7	139.9	141.6	1.31	(1.05–1.56)	25,075
Mean DBP (mmHg)	84.2	84.5	85.2	85.5	86.2	0.64	(0.50–0.77)	25,075
Mean age at child's birth (years)	30.4	30.2	30.4	30.5	30.5	0.01	(-0.06–0.09)	25,330
Proportion ever smoked (%)	63.2	61.9	64.1	64.5	64.1	1.01	(0.98–1.04)	21,570
Proportion drinking ≥ 5 times fortnightly (%)	8.4	8.0	7.8	7.7	8.3	0.99	(0.94–1.04)	21,127
Proportion educated ≥10 years (%)	56.5	53.4	50.3	48.3	46.0	0.88	(0.85–0.90)	20,538

Proportion physically active (%)	87.1	86.3	86.0	83.9	84.4	0.93	(0.89–0.97)	17,603
Proportion taking antihypertensives (%)	8.9	9.6	9.6	9.8	9.8	1.02	(0.98–1.06)	25,058

BMI: body mass index, CI: confidence interval, DBP: diastolic blood pressure, OR: odds ratio, SBP: systolic blood pressure, SD: standard deviation

**adjusted for age, sex and HUNT survey*

†*Continuous variables are represented as regression coefficients and proportions are represented as ORs per SD increase in mother's SBP*

Supplementary Table S4. Properties of offspring and parents according to quintiles of mother's DBP

Subject, Measurement	Quintile of mother's DBP					Linear or logistic regression per SD*		
	1st	2nd	3rd	4th	5th	Regression Coefficient or OR†	95% CI	N
**Offspring**								
Mean BMI (kg/m^2)	23.8	23.8	24.1	24.0	24.4	0.19	(0.15–0.24)	32,107
Mean SBP (mmHg)	123.6	124.8	125.6	126.7	128.5	1.68	(1.52–1.84)	32,227
Mean DBP (mmHg)	71.9	73.3	74.1	75.2	76.6	1.55	(1.42–1.68)	32,227
Proportion ever smoked (%)	37.5	37.4	38.2	38.7	39.4	1.01	(0.99–1.04)	29,572
Proportion drinking ≥ 5 times fortnightly (%)	4.5	3.9	3.1	4.4	3.7	0.94	(0.88–1.01)	20,690
Proportion educated ≥ 10 years (%)	76.3	75.5	74.4	74.8	73.3	0.96	(0.93–0.99)	17,078
Proportion physically active (%)	91.1	91.3	91.3	90.1	91.2	1.00	(0.95–1.05)	18,255
Proportion taking antihypertensives (%)	2.3	2.7	2.6	3.7	4.0	1.23	(1.14–1.31)	23,738
**Mothers**								
Mean BMI (kg/m^2)	23.9	24.7	25.1	25.9	27.0	1.09	(1.04–1.14)	32,018

Mean SBP (mmHg)	120.3	127.9	133.7	140.5	153.5	12.07	(11.83–12.31)	32,227
Mean DBP (mmHg)	68.7	76.9	82.5	88.4	98.8	10.86	(10.78–10.95)	32,227
Mean age at child's birth (years)	27.4	27.4	27.4	27.4	27.2	-0.09	(-0.16–-0.03)	32,227
Proportion ever smoked (%)	51.9	48.4	46.4	44.7	43.2	0.88	(0.86–0.91)	27,695
Proportion drinking ≥ 5 times fortnightly (%)	3.2	2.7	2.5	2.8	2.7	0.93	(0.86–1.00)	27,107
Proportion educated ≥10 years (%)	48.4	45.6	43.2	41.4	39.4	0.91	(0.88–0.93)	26,534
Proportion physically active (%)	85.8	86.7	84.9	84.8	83.0	0.92	(0.89–0.96)	22,955
Proportion taking antihypertensives (%)	5.6	9.0	13.2	18.7	26.9	1.89	(1.83–1.95)	32,227
**Fathers**								
Mean BMI (kg/m^2)	25.3	25.4	25.5	25.5	25.7	0.13	(0.08–0.17)	25,008
Mean SBP (mmHg)	136.6	138.0	138.4	139.2	140.5	1.22	(0.98–1.47)	25,075
Mean DBP (mmHg)	84.3	85.2	85.6	86.1	86.9	0.80	(0.65–0.95)	25,075
Mean age at child's birth (years)	30.4	30.3	30.5	30.5	30.4	0.00	(-0.08–0.08)	25,330
Proportion ever smoked (%)	63.3	62.8	63.7	63.5	64.5	1.00	(0.98–1.03)	21,570
Proportion drinking ≥ 5 times fortnightly (%)	8.0	8.0	7.5	7.8	9.0	1.01	(0.96–1.07)	21,127
Proportion educated ≥10 years (%)	56.2	52.7	50.3	49.0	46.0	0.89	(0.86–0.91)	20,538

Proportion physically active (%)	86.5	86.6	85.6	84.7	84.3	0.93	(0.89–0.97)	17,603
Proportion taking antihypertensives (%)	8.7	9.9	9.6	9.8	9.7	1.01	(0.96–1.05)	25,058

BMI: body mass index, CI: confidence interval, DBP: diastolic blood pressure, OR: odds ratio, SBP: systolic blood pressure, SD: standard deviation

**adjusted for age, sex and HUNT survey*

†*Continuous variables are represented as regression coefficients and proportions are represented as ORs per SD increase in mother's DBP*

Supplementary Table S5. Properties of offspring and parents according to quintiles of father's SBP

Subject, Measurement	Quintile of father's SBP					Linear or logistic regression per SD*		
	1st	2nd	3rd	4th	5th	Regression Coefficient or OR†	95% CI	N
**Offspring**								
Mean BMI (kg/m²)	23.7	23.8	24.0	24.0	24.2	0.14	(0.09–0.18)	27,429
Mean SBP (mmHg)	123.0	124.3	125.5	126.6	128.5	1.74	(1.59–1.90)	27,535
Mean DBP (mmHg)	71.7	72.6	73.5	73.9	75.2	1.07	(0.95–1.19)	27,535
Proportion ever smoked (%)	35.1	37.1	36.0	35.8	37.5	1.01	(0.98–1.03)	25,490
Proportion drinking ≥ 5 times fortnightly (%)	3.5	3.9	4.0	3.5	3.3	0.95	(0.89–1.02)	17,487
Proportion educated ≥10 years (%)	79.3	78.8	78.6	76.9	77.1	0.97	(0.94–1.01)	14,159
Proportion physically active (%)	92.0	91.6	91.9	92.4	91.2	0.98	(0.94–1.03)	15,606
Proportion taking antihypertensives (%)	1.9	1.7	3.0	2.4	3.4	1.17	(1.09–1.26)	19,882
**Mothers**								
Mean BMI (kg/m²)	24.8	24.9	25.1	25.0	25.4	0.19	(0.14–0.25)	25,149

Mean SBP (mmHg)	130.3	132.1	133.2	133.7	135.3	1.61	(1.33–1.90)	25,230
Mean DBP (mmHg)	79.8	80.8	81.3	81.5	82.4	0.75	(0.61–0.89)	25,230
Mean age at child's birth (years)	27.3	27.2	27.2	27.1	27.2	-0.01	(-0.07–0.06)	25,411
Proportion ever smoked (%)	49.8	49.5	48.0	49.9	48.6	0.98	(0.96–1.01)	21,753
Proportion drinking ≥ 5 times fortnightly (%)	2.8	2.6	3.1	2.9	2.9	1.02	(0.95–1.10)	21,353
Proportion educated ≥10 years (%)	51.6	48.6	47.3	47.0	43.6	0.93	(0.90–0.95)	21,113
Proportion physically active (%)	88.2	87.3	88.1	86.3	86.6	0.96	(0.92–1.00)	17,805
Proportion taking antihypertensives (%)	10.0	11.5	12.4	12.5	12.8	1.09	(1.06–1.13)	25,216
**Fathers**								
Mean BMI (kg/m^2)	24.5	25.2	25.6	25.9	26.3	0.51	(0.48–0.55)	27,424
Mean SBP (mmHg)	118.6	130.5	139.2	148.7	168.6	17.22	(17.11–17.34)	27,535
Mean DBP (mmHg)	76.8	81.9	85.4	88.6	94.9	5.93	(5.82–6.03)	27,535
Mean age at child's birth (years)	30.6	30.4	30.4	30.3	30.5	-0.05	(-0.12–0.02)	27,535
Proportion ever smoked (%)	64.7	63.7	62.4	62.8	63.0	0.97	(0.95–0.99)	23,697
Proportion drinking ≥ 5 times fortnightly (%)	7.5	8.2	7.7	7.9	9.0	1.03	(0.99–1.08)	23,160
Proportion educated ≥10 years (%)	53.8	50.7	50.4	47.7	45.1	0.91	(0.88–0.93)	22,438

Proportion physically active (%)	85.9	84.4	85.8	84.7	84.0	0.99	(0.95–1.02)	19,420
Proportion taking antihypertensives (%)	4.4	6.4	9.4	12.1	19.3	1.68	(1.62–1.74)	27,535

BMI: body mass index, CI: confidence interval, DBP: diastolic blood pressure, OR: odds ratio, SBP: systolic blood pressure, SD: standard deviation

**adjusted for age, sex and HUNT survey*

†*Continuous variables are represented as regression coefficients and proportions are represented as ORs per SD increase in father's SBP*

Supplementary Table S6. Properties of offspring and parents according to quintiles of father's DBP

	Quintile of father's DBP					Linear or logistic regression per SD*		
Subject, Measurement	1st	2nd	3rd	4th	5th	Regression Coefficient or OR†	95% CI	N
**Offspring**								
Mean BMI (kg/m^2)	23.8	23.8	23.9	24.0	24.2	0.14	(0.09–0.19)	27,429
Mean SBP (mmHg)	123.6	124.4	125.5	126.0	127.4	1.32	(1.16–1.48)	27,535
Mean DBP (mmHg)	71.7	72.3	73.7	74.1	75.7	1.37	(1.24–1.51)	27,535
Proportion ever smoked (%)	35.8	35.5	36.6	36.5	37.2	1.01	(0.99–1.04)	25,490
Proportion drinking ≥ 5 times fortnightly (%)	3.8	3.5	3.9	3.3	3.8	1.00	(0.93–1.08)	17,487
Proportion educated ≥10 years (%)	79.5	78.1	78.3	77.2	77.6	0.96	(0.93–1.00)	14,159
Proportion physically active (%)	91.5	92.3	91.5	92.2	91.6	0.99	(0.94–1.05)	15,606
Proportion taking antihypertensives (%)	1.9	2.1	2.6	2.7	3.3	1.21	(1.12–1.32)	19,882
**Mothers**								
Mean BMI (kg/m^2)	24.8	24.8	25.1	25.1	25.4	0.18	(0.12–0.23)	25,149

Mean SBP (mmHg)	130.2	130.7	132.3	131.8	133.6	1.10	(0.82–1.39)	25,230
Mean DBP (mmHg)	80.5	81.2	82.0	81.9	83.1	0.80	(0.65–0.96)	25,230
Mean age at child's birth (years)	27.1	27.1	27.3	27.2	27.3	0.04	(-0.03–0.10)	25,411
Proportion ever smoked (%)	49.7	50.1	47.7	49.0	49.1	0.99	(0.96–1.02)	21,753
Proportion drinking ≥ 5 times fortnightly (%)	2.9	2.5	3.1	3.1	2.7	1.00	(0.93–1.09)	21,353
Proportion educated ≥10 years (%)	49.1	49.9	47.9	46.8	44.3	0.95	(0.92–0.97)	21,113
Proportion physically active (%)	88.2	87.4	87.7	87.8	85.2	0.92	(0.88–0.96)	17,805
Proportion taking antihypertensives (%)	11.4	10.8	12.4	11.7	12.9	1.05	(1.01–1.09)	25,216
**Fathers**								
Mean BMI (kg/m^2)	24.4	25.0	25.5	26.0	26.7	0.80	(0.76–0.83)	27,424
Mean SBP (mmHg)	126.7	133.0	138.5	144.7	156.1	10.63	(10.43–10.82)	27,535
Mean DBP (mmHg)	72.1	80.3	86.0	91.4	101.9	10.56	(10.51–10.62)	27,535
Mean age at child's birth (years)	30.6	30.3	30.6	30.4	30.4	-0.06	(-0.14–0.01)	27,535
Proportion ever smoked (%)	66.5	64.0	62.7	61.8	61.1	0.92	(0.89–0.94)	23,697
Proportion drinking ≥ 5 times fortnightly (%)	7.8	7.5	8.3	7.7	9.0	1.05	(1.00–1.10)	23,160
Proportion educated ≥10 years (%)	51.4	51.8	49.7	48.8	46.3	0.94	(0.91–0.96)	22,438

Proportion physically active (%)	85.7	84.9	85.5	84.7	83.9	0.97	(0.93–1.00)	19,420
Proportion taking antihypertensives (%)	4.1	4.9	8.3	12.6	22.1	2.01	(1.93–2.09)	27,535

BMI: body mass index, CI: confidence interval, DBP: diastolic blood pressure, OR: odds ratio, SBP: systolic blood pressure, SD: standard deviation

*adjusted for age, sex and HUNT survey

†*Continuous variables are represented as regression coefficients and proportions are represented as ORs per SD increase in father's DBP*

Supplementary Table S7. Adjusted HRs per SD of offspring SBP for combined parents, and estimates of HR for own SBP estimated by conventional observational and IV analyses

Cause of Death	Deaths	Offspring SBP, fully adjusted*			Own SBP, fully adjusted			IV, fully adjusted			P-value for comparison with own SBP
		HR	95% CI	P-value	HR	95% CI	P-value	HR	95% CI	P-value	
Diabetes	312	1.16	(1.03–1.32)	0.02	1.33	(1.23–1.45)	<0.0001	2.00	(1.12–3.55)	0.02	0.15
Respiratory Diseases	1,418	1.01	(0.96–1.08)	0.64	0.98	(0.94–1.02)	0.28	1.07	(0.81–1.40)	0.64	0.51
Cancer	4,566	1.04	(1.00–1.08)	0.03	1.02	(1.00–1.05)	0.07	1.20	(1.02–1.42)	0.03	0.05
Lung Cancer	695	0.95	(0.87–1.05)	0.31	1.00	(0.94–1.07)	0.97	0.80	(0.52–1.23)	0.31	0.31
Colorectal Cancer	704	1.03	(0.94–1.12)	0.54	0.97	(0.91–1.03)	0.32	1.13	(0.76–1.69)	0.54	0.46
Pancreatic Cancer	273	1.17	(1.01–1.35)	0.04	1.00	(0.92–1.09)	0.99	2.06	(1.05–4.02)	0.04	0.04
Stomach Cancer	257	0.95	(0.81–1.11)	0.50	1.01	(0.91–1.11)	0.90	0.78	(0.37–1.63)	0.50	0.49
External Causes	691	1.02	(0.93–1.12)	0.64	1.06	(1.00–1.13)	0.06	1.11	(0.72–1.70)	0.64	0.84

CI: confidence interval, HR: hazard ratio, IV: instrumental variable, SBP: systolic blood pressure, SD: standard deviation

**adjusted for age, sex and HUNT survey of offspring/parent from whom SBP was measured, offspring and parental smoking status, age, DOB, alcohol consumption, education, own and spouse's employment, exercise and sex of parent*

Supplementary Table S8. Adjusted HRs per SD of offspring DBP for combined parents, and estimates of HR for own DBP estimated by conventional observational and IV analyses

Cause of Death	Deaths	Offspring DBP, fully adjusted*			Own DBP, fully adjusted			IV, fully adjusted			P-value for comparison with own DBP
		HR	95% CI	P-value	HR	95% CI	P-value	HR	95% CI	P-value	
Diabetes	312	1.11	(0.99–1.25)	0.08	1.35	(1.22–1.51)	<0.0001	1.83	(0.92–3.62)	0.08	0.39
Respiratory Diseases	1,418	1.01	(0.96–1.07)	0.64	0.95	(0.91–1.01)	0.08	1.08	(0.79–1.47)	0.64	0.45
Cancer	4,566	1.02	(0.99–1.06)	0.16	1.01	(0.98–1.04)	0.37	1.14	(0.95–1.37)	0.16	0.19
Lung Cancer	695	1.03	(0.95–1.12)	0.52	0.95	(0.88–1.02)	0.17	1.17	(0.72–1.91)	0.52	0.37
Colorectal Cancer	704	1.01	(0.93–1.09)	0.86	1.01	(0.94–1.09)	0.70	1.04	(0.66–1.64)	0.86	0.91
Pancreatic Cancer	273	1.11	(0.98–1.27)	0.11	1.04	(0.93–1.17)	0.46	1.86	(0.87–3.97)	0.11	0.14
Stomach Cancer	257	0.90	(0.78–1.03)	0.12	1.02	(0.91–1.15)	0.71	0.54	(0.24–1.19)	0.12	0.11
External Causes	691	1.05	(0.97–1.14)	0.21	1.08	(1.00–1.16)	0.05	1.33	(0.85–2.10)	0.21	0.36

CI: confidence interval, DBP: diastolic blood pressure, HR: hazard ratio, IV: instrumental variable, SD: standard deviation

**adjusted for age, sex and HUNT survey of offspring/parent from whom DBP was measured, offspring and parental smoking status, age, DOB, alcohol consumption, education, own and spouse's employment, exercise and sex of parent*

Supplementary Table S9. Adjusted HRs per SD of offspring SBP for mothers, and estimates of HR for own SBP estimated by conventional observational and IV analyses

Cause of Death	Deaths	Offspring SBP, fully adjusted*			Own SBP, fully adjusted			IV, fully adjusted			P-value for comparison with own SBP
		HR	95% CI	P-value	HR	95% CI	P-value	HR	95% CI	P-value	
All cause	8,907	1.04	(1.02–1.06)	0.001	1.11	(1.10–1.13)	<0.0001	1.19	(1.07–1.33)	0.001	0.21
Cardiovascular Disease	4,177	1.05	(1.01–1.08)	0.01	1.20	(1.17–1.22)	<0.0001	1.24	(1.06–1.44)	0.01	0.67
Coronary Heart Disease	2,749	1.02	(0.98–1.06)	0.32	1.16	(1.13–1.19)	<0.0001	1.10	(0.91–1.32)	0.32	0.55
Stroke	1,315	1.04	(0.98–1.10)	0.25	1.21	(1.16–1.25)	<0.0001	1.17	(0.89–1.54)	0.25	0.82
Diabetes	183	1.18	(1.01–1.38)	0.04	1.32	(1.19–1.46)	<0.0001	2.10	(1.02–4.31)	0.04	0.19
Respiratory Diseases	671	0.98	(0.90–1.06)	0.58	0.96	(0.91–1.02)	0.19	0.90	(0.61–1.31)	0.58	0.70
Cancer	2,055	1.04	(0.99–1.10)	0.13	1.03	(1.00–1.07)	0.06	1.20	(0.95–1.52)	0.13	0.23
Lung Cancer	256	0.94	(0.80–1.10)	0.44	0.95	(0.86–1.06)	0.40	0.76	(0.37–1.54)	0.44	0.49
Breast Cancer	281	1.14	(0.99–1.31)	0.07	1.02	(0.92–1.12)	0.72	1.78	(0.95–3.36)	0.07	0.10
Ovarian Cancer	125	1.13	(0.91–1.40)	0.27	1.02	(0.88–1.18)	0.82	1.72	(0.66–4.51)	0.27	0.26

Colorectal Cancer	362	0.97	(0.86–1.10)	0.64	0.97	(0.89–1.05)	0.45	0.87	(0.50–1.54)	0.64		0.71
Pancreatic Cancer	120	1.04	(0.83–1.29)	0.75	0.95	(0.82–1.11)	0.54	1.17	(0.44–3.13)	0.75		0.65
Stomach Cancer	103	1.02	(0.81–1.27)	0.88	1.01	(0.87–1.17)	0.93	1.08	(0.40–2.95)	0.88		0.90
External Causes	296	1.01	(0.89–1.15)	0.86	1.06	(0.97–1.16)	0.21	1.05	(0.59–1.89)	0.86		0.99

CI: confidence interval, HR: hazard ratio, IV: instrumental variable, SBP: systolic blood pressure, SD: standard deviation

**adjusted for age, sex and HUNT survey of offspring/parent from whom SBP was measured, offspring and parental smoking status, age, DOB, alcohol consumption, education, own and spouse's employment, exercise and sex of parent*

Supplementary Table S10. Adjusted HRs per SD of offspring DBP for mothers, and estimates of HR for own DBP estimated by conventional observational and IV analyses

Cause of Death	Deaths	Offspring DBP, fully adjusted*			Own DBP, fully adjusted			IV, fully adjusted			P-value for comparison with own DBP
		HR	95% CI	P-value	HR	95% CI	P-value	HR	95% CI	P-value	
All cause	8,907	1.03	(1.01–1.05)	0.01	1.10	(1.08–1.12)	<0.0001	1.19	(1.05–1.35)	0.01	0.20
Cardiovascular Disease	4,177	1.04	(1.01–1.07)	0.02	1.18	(1.15–1.22)	<0.0001	1.24	(1.03–1.48)	0.02	0.62
Coronary Heart Disease	2,749	1.03	(0.99–1.07)	0.20	1.14	(1.10–1.17)	<0.0001	1.15	(0.93–1.43)	0.21	0.89
Stroke	1,315	1.04	(0.98–1.10)	0.17	1.23	(1.17–1.29)	<0.0001	1.25	(0.91–1.72)	0.17	0.91
Diabetes	183	1.12	(0.96–1.30)	0.14	1.42	(1.25–1.61)	<0.0001	1.89	(0.80–4.43)	0.14	0.52
Respiratory Diseases	671	0.98	(0.91–1.06)	0.63	0.95	(0.89–1.03)	0.20	0.90	(0.57–1.40)	0.63	0.77
Cancer	2,055	1.03	(0.98–1.08)	0.20	1.02	(0.98–1.06)	0.35	1.19	(0.91–1.55)	0.20	0.26
Lung Cancer	256	1.03	(0.90–1.18)	0.65	0.86	(0.76–0.98)	0.02	1.19	(0.56–2.52)	0.65	0.40
Breast Cancer	281	0.98	(0.86–1.11)	0.75	1.01	(0.90–1.13)	0.89	0.89	(0.43–1.83)	0.75	0.74
Ovarian Cancer	125	1.09	(0.90–1.31)	0.37	0.95	(0.79–1.13)	0.54	1.63	(0.56–4.73)	0.37	0.24

Colorectal Cancer	362	0.98	(0.88–1.10)	0.78	1.01	(0.91–1.11)	0.91	0.92	(0.49–1.72)	0.78		0.77
Pancreatic Cancer	120	1.01	(0.84–1.23)	0.90	1.01	(0.85–1.20)	0.89	1.07	(0.36–3.18)	0.90		0.92
Stomach Cancer	103	0.86	(0.70–1.06)	0.16	1.00	(0.83–1.21)	0.98	0.42	(0.13–1.40)	0.16		0.19
External Causes	296	1.05	(0.93–1.19)	0.40	1.05	(0.95–1.17)	0.34	1.34	(0.68–2.65)	0.40		0.49

CI: confidence interval, DBP: diastolic blood pressure, HR: hazard ratio, IV: instrumental variable, SD: standard deviation

**adjusted for age, sex and HUNT survey of offspring/parent from whom DBP was measured, offspring and parental smoking status, age, DOB, alcohol consumption, education, own and spouse's employment, exercise and sex of parent*

Supplementary Table S11. Adjusted HRs per SD of offspring SBP for fathers, and estimates of HR for own SBP estimated by conventional observational and IV analyses

Cause of Death	Deaths	Offspring SBP, fully adjusted*			Own SBP, fully adjusted			IV, fully adjusted			P-value for comparison with own SBP
		HR	95% CI	P-value	HR	95% CI	P-value	HR	95% CI	P-value	
All cause	9,633	1.07	(1.04–1.09)	<0.0001	1.10	(1.08–1.12)	<0.0001	1.36	(1.21–1.52)	<0.0001	0.001
Cardiovascular Disease	4,595	1.09	(1.05–1.13)	<0.0001	1.18	(1.16–1.21)	<0.0001	1.50	(1.27–1.76)	<0.0001	0.005
Coronary Heart Disease	3,822	1.09	(1.05–1.14)	<0.0001	1.14	(1.11–1.16)	<0.0001	1.53	(1.28–1.82)	<0.0001	0.001
Stroke	953	1.05	(0.98–1.14)	0.18	1.24	(1.19–1.30)	<0.0001	1.28	(0.90–1.82)	0.18	0.88
Diabetes	129	1.13	(0.92–1.39)	0.24	1.35	(1.20–1.53)	<0.0001	1.77	(0.68–4.64)	0.24	0.62
Respiratory Diseases	747	1.06	(0.98–1.16)	0.14	0.99	(0.94–1.05)	0.82	1.34	(0.90–1.99)	0.14	0.13
Cancer	2,511	1.04	(0.99–1.09)	0.11	1.01	(0.98–1.04)	0.52	1.21	(0.96–1.52)	0.11	0.13
Lung Cancer	439	0.95	(0.84–1.08)	0.47	1.02	(0.94–1.11)	0.59	0.81	(0.45–1.44)	0.47	0.43
Prostate Cancer	526	1.05	(0.95–1.16)	0.35	1.00	(0.94–1.07)	0.91	1.26	(0.77–2.04)	0.35	0.38
Colorectal Cancer	342	1.10	(0.96–1.25)	0.17	0.97	(0.89–1.06)	0.50	1.53	(0.83–2.83)	0.17	0.14

Pancreatic Cancer	153	1.28	(1.07–1.55)	0.01	1.04	(0.92–1.19)	0.53	3.24	(1.35–7.75)	0.01	0.02
Stomach Cancer	154	0.89	(0.73–1.09)	0.27	1.01	(0.89–1.15)	0.87	0.58	(0.22–1.51)	0.27	0.25
External Causes	395	1.02	(0.90–1.15)	0.80	1.06	(0.97–1.15)	0.18	1.08	(0.60–1.93)	0.80	0.95

CI: confidence interval, HR: hazard ratio, IV: instrumental variable, SBP: systolic blood pressure, SD: standard deviation

adjusted for age, sex and HUNT survey of offspring/parent from whom SBP was measured, offspring and parental smoking status, age, DOB, alcohol consumption, education, own and spouse's employment, exercise and sex of parent

Supplementary Table S12. Adjusted HRs per SD of offspring DBP for fathers, and estimates of HR for own DBP estimated by conventional observational and IV analyses

Cause of Death	Deaths	Offspring DBP, fully adjusted*			Own DBP, fully adjusted			IV, fully adjusted			P-value for comparison with own DBP
		HR	95% CI	P-value	HR	95% CI	P-value	HR	95% CI	P-value	
All cause	9,633	1.06	(1.04–1.08)	<0.0001	1.10	(1.08–1.12)	<0.0001	1.41	(1.24–1.61)	<0.0001	<0.001
Cardiovascular Disease	4,595	1.09	(1.06–1.13)	<0.0001	1.19	(1.16–1.22)	<0.0001	1.68	(1.40–2.02)	<0.0001	<0.001
Coronary Heart Disease	3,822	1.08	(1.04–1.12)	<0.0001	1.14	(1.10–1.17)	<0.0001	1.58	(1.29–1.93)	<0.0001	<0.001
Stroke	953	1.05	(0.99–1.13)	0.13	1.32	(1.25–1.40)	<0.0001	1.37	(0.92–2.03)	0.13	0.86
Diabetes	129	1.08	(0.90–1.30)	0.39	1.27	(1.08–1.49)	0.003	1.61	(0.54–4.79)	0.39	0.69
Respiratory Diseases	747	1.05	(0.97–1.13)	0.22	0.96	(0.89–1.02)	0.19	1.33	(0.85–2.09)	0.22	0.16
Cancer	2,511	1.02	(0.97–1.06)	0.47	1.01	(0.97–1.04)	0.77	1.10	(0.85–1.41)	0.47	0.48
Lung Cancer	439	1.03	(0.93–1.14)	0.59	0.99	(0.90–1.08)	0.83	1.18	(0.64–2.17)	0.59	0.59
Prostate Cancer	526	0.98	(0.89–1.07)	0.60	0.99	(0.91–1.07)	0.74	0.86	(0.50–1.49)	0.60	0.64
Colorectal Cancer	342	1.03	(0.92–1.15)	0.64	1.02	(0.92–1.13)	0.67	1.17	(0.60–2.31)	0.64	0.69

Pancreatic Cancer	153	1.19	(1.01–1.41)	0.04	1.07	(0.92–1.24)	0.37	2.85	(1.05–7.72)	0.04	0.06
Stomach Cancer	154	0.93	(0.78–1.10)	0.40	1.04	(0.89–1.20)	0.65	0.65	(0.23–1.80)	0.40	0.36
External Causes	395	1.04	(0.93–1.16)	0.47	1.10	(1.00–1.20)	0.05	1.27	(0.67–2.40)	0.47	0.65

CI: confidence interval, DBP: diastolic blood pressure, HR: hazard ratio, IV: instrumental variable, SD: standard deviation

adjusted for age, sex and HUNT survey of offspring/parent from whom DBP was measured, offspring and parental smoking status, age, DOB, alcohol consumption, education, own and spouse's employment, exercise and sex of parent

Supplementary Table S13. HRs for combined parents' SBP estimated with fully adjusted and fully adjusted+BMI IV models

Cause of Death	Deaths	Offspring SBP, fully adjusted*+BMI			Own SBP, fully adjusted*+BMI			IV, fully adjusted*			IV, fully adjusted*+BMI		
		HR	95% CI	P-value	HR	95% CI	P-value	HR	95% CI	P-value	HR	95% CI	P-value
All cause	18,540	1.05	(1.04–1.07)	<0.0001	1.11	(1.10–1.12)	<0.0001	1.28	(1.18–1.39)	<0.0001	1.30	(1.19–1.41)	<0.0001
Cardiovascular Disease	8,772	1.07	(1.04–1.09)	<0.0001	1.19	(1.17–1.21)	<0.0001	1.38	(1.23–1.54)	<0.0001	1.37	(1.22–1.55)	<0.0001
Coronary Heart Disease	6,571	1.06	(1.04–1.09)	<0.0001	1.15	(1.13–1.18)	<0.0001	1.32	(1.17–1.50)	<0.0001	1.36	(1.19–1.55)	<0.0001
Stroke	2,268	1.04	(0.99–1.09)	0.13	1.23	(1.19–1.27)	<0.0001	1.21	(0.98–1.51)	0.08	1.20	(0.95–1.51)	0.13
Diabetes	312	1.13	(0.99–1.28)	0.07	1.30	(1.19–1.41)	<0.0001	2.00	(1.12–3.55)	0.02	1.79	(0.95–3.36)	0.07
Respiratory Diseases	1,418	1.04	(0.98–1.10)	0.22	1.02	(0.98–1.06)	0.42	1.07	(0.81–1.40)	0.64	1.20	(0.90–1.61)	0.22
Cancer	4,566	1.04	(1.01–1.08)	0.02	1.02	(1.00–1.05)	0.08	1.20	(1.02–1.42)	0.03	1.23	(1.03–1.46)	0.02
Lung Cancer	695	0.96	(0.87–1.05)	0.37	1.02	(0.96–1.09)	0.51	0.80	(0.52–1.23)	0.31	0.81	(0.52–1.28)	0.37

Colorectal Cancer	704	1.03	(0.94–1.12)	0.56	0.96	(0.91–1.03)	0.25	1.13	(0.76–1.69)	0.54	1.13	(0.74–1.73)	0.56
Pancreatic Cancer	273	1.17	(1.01–1.35)	0.04	0.99	(0.91–1.09)	0.91	2.06	(1.05–4.02)	0.04	2.10	(1.03–4.31)	0.04
Stomach Cancer	257	0.95	(0.81–1.12)	0.53	1.00	(0.91–1.11)	0.95	0.78	(0.37–1.63)	0.50	0.78	(0.35–1.70)	0.53
External Causes	691	1.04	(0.95–1.15)	0.36	1.08	(1.02–1.14)	0.01	1.11	(0.72–1.70)	0.64	1.24	(0.79–1.94)	0.36

BMI: body mass index, CI: confidence interval, HR: hazard ratio, IV: instrumental variable, SBP: systolic blood pressure, SD: standard deviation

**adjusted for age, sex and HUNT survey of offspring/parent from whom SBP was measured, offspring and parental smoking status, age, DOB, alcohol consumption, education, own and spouse's employment, exercise and sex of parent*

Supplementary Table S14. HRs for combined parents DBP estimated with fully adjusted and fully adjusted+BMI IV models

Cause of Death	Deaths	Offspring DBP, fully adjusted*+BMI			Own DBP, fully adjusted*+BMI			IV, fully adjusted*			IV, fully adjusted*+BMI		
		HR	95% CI	P-value	HR	95% CI	P-value	HR	95% CI	P-value	HR	95% CI	P-value
All cause	18,540	1.05	(1.03–1.06)	<0.0001	1.10	(1.09–1.12)	<0.0001	1.31	(1.19–1.43)	<0.0001	1.33	(1.20–1.46)	<0.0001
Cardiovascular Disease	8,772	1.06	(1.04–1.09)	<0.0001	1.17	(1.15–1.20)	<0.0001	1.46	(1.28–1.66)	<0.0001	1.46	(1.27–1.68)	<0.0001
Coronary Heart Disease	6,571	1.06	(1.03–1.09)	<0.0001	1.14	(1.11–1.16)	<0.0001	1.38	(1.19–1.59)	<0.0001	1.43	(1.22–1.67)	<0.0001
Stroke	2,268	1.04	(1.00–1.09)	0.05	1.26	(1.21–1.32)	<0.0001	1.30	(1.02–1.66)	0.03	1.30	(1.00–1.69)	0.05
Diabetes	312	1.09	(0.97–1.23)	0.16	1.27	(1.13–1.41)	<0.0001	1.83	(0.92–3.62)	0.08	1.72	(0.81–3.63)	0.16
Respiratory Diseases	1,418	1.03	(0.98–1.09)	0.25	1.01	(0.96–1.06)	0.74	1.08	(0.79–1.47)	0.64	1.22	(0.87–1.70)	0.25
Cancer	4,566	1.02	(0.99–1.05)	0.18	1.01	(0.98–1.04)	0.41	1.14	(0.95–1.37)	0.16	1.15	(0.94–1.39)	0.18
Lung Cancer	695	1.03	(0.95–1.12)	0.45	0.98	(0.91–1.06)	0.59	1.17	(0.72–1.91)	0.52	1.23	(0.73–2.07)	0.44

Colorectal Cancer	704	1.00	(0.93–1.08)	0.94	1.01	(0.93–1.09)	0.87	1.04	(0.66–1.64)	0.86	1.02	(0.62–1.66)	0.94
Pancreatic Cancer	273	1.11	(0.97–1.26)	0.13	1.04	(0.92–1.16)	0.56	1.86	(0.87–3.97)	0.11	1.89	(0.83–4.27)	0.13
Stomach Cancer	257	0.90	(0.78–1.03)	0.13	1.02	(0.90–1.15)	0.74	0.54	(0.24–1.19)	0.12	0.52	(0.22–1.21)	0.13
External Causes	691	1.06	(0.98–1.15)	0.12	1.12	(1.04–1.21)	0.004	1.33	(0.85–2.10)	0.21	1.47	(0.91–2.40)	0.12

BMI: body mass index, CI: confidence interval, DBP: diastolic blood pressure, HR: hazard ratio, IV: instrumental variable, SD: standard deviation

**adjusted for age, sex and HUNT survey of offspring/parent from whom DBP was measured, offspring and parental smoking status, age, DOB, alcohol consumption, education, own and spouse's employment, exercise and sex of parent*

Supplementary Table S15. Adjusted HRs per SD of offspring SBP for combined parents, and estimates of HR for own SBP estimated by conventional observational and IV analyses with exclusion of individuals taking antihypertensive medication

Cause of Death	Deaths	Offspring SBP, fully adjusted*			Own SBP, fully adjusted*			IV, fully adjusted*			P-value for comparison with own SBP
		HR	95% CI	P-value	HR	95% CI	P-value	HR	95% CI	P-value	
All cause	13,418	1.05	(1.03–1.07)	<0.0001	1.09	(1.08–1.11)	<0.0001	1.26	(1.13–1.40)	<0.0001	0.01
Cardiovascular Disease	5,811	1.05	(1.02–1.09)	0.002	1.18	(1.16–1.21)	<0.0001	1.28	(1.09–1.50)	0.002	0.31
Coronary Heart Disease	4,437	1.06	(1.02–1.10)	0.001	1.14	(1.12–1.17)	<0.0001	1.33	(1.12–1.59)	0.001	0.07
Stroke	1,465	1.04	(0.98–1.11)	0.20	1.21	(1.17–1.26)	<0.0001	1.22	(0.90–1.66)	0.20	0.96
Diabetes	192	1.23	(1.03–1.47)	0.02	1.32	(1.17–1.48)	<0.0001	2.78	(1.18–6.56)	0.02	0.08
Respiratory Diseases	1,095	1.03	(0.96–1.11)	0.37	0.98	(0.93–1.03)	0.43	1.18	(0.82–1.69)	0.37	0.31
Cancer	3,687	1.06	(1.01–1.10)	0.01	1.02	(0.99–1.05)	0.13	1.31	(1.07–1.61)	0.01	0.01
Lung Cancer	612	0.93	(0.84–1.03)	0.16	1.00	(0.94–1.07)	0.95	0.70	(0.42–1.16)	0.17	0.16
Colorectal Cancer	543	1.07	(0.97–1.20)	0.19	0.94	(0.87–1.01)	0.09	1.42	(0.84–2.41)	0.19	0.11
Pancreatic Cancer	210	1.22	(1.04–1.43)	0.01	0.99	(0.89–1.11)	0.90	2.66	(1.21–5.81)	0.01	0.01

Stomach Cancer	193	0.95	(0.78–1.16)	0.61	0.95	(0.84–1.07)	0.39	0.78	(0.29–2.05)	0.61	0.70
External Causes	553	1.05	(0.95–1.17)	0.32	1.06	(0.98–1.13)	0.13	1.30	(0.77–2.17)	0.32	0.43

BMI: body mass index, CI: confidence interval, HR: hazard ratio, IV: instrumental variable, SBP: systolic blood pressure, SD: standard deviation

**adjusted for age, sex and HUNT survey of offspring/parent from whom SBP was measured, offspring and parental smoking status, age, DOB, alcohol consumption, education, own and spouse's employment, exercise and sex of parent*

Supplementary Table S16. Adjusted HRs per SD of offspring DBP for combined parents, and estimates of HR for own DBP estimated by conventional observational and IV analyses with exclusion of individuals taking antihypertensive medication

Cause of Death	Deaths	Offspring DBP, fully adjusted*			Own DBP, fully adjusted*			IV, fully adjusted*			P-value for comparison with own DBP
		HR	95% CI	P-value	HR	95% CI	P-value	HR	95% CI	P-value	
All cause	13,418	1.04	(1.02–1.06)	<0.0001	1.08	(1.06–1.10)	<0.0001	1.29	(1.14–1.45)	<0.0001	0.003
Cardiovascular Disease	5,811	1.05	(1.02–1.08)	0.001	1.17	(1.14–1.20)	<0.0001	1.33	(1.12–1.59)	0.002	0.15
Coronary Heart Disease	4,437	1.05	(1.02–1.09)	0.002	1.13	(1.10–1.16)	<0.0001	1.37	(1.12–1.67)	0.002	0.05
Stroke	1,465	1.04	(0.98–1.10)	0.16	1.25	(1.19–1.31)	<0.0001	1.28	(0.90–1.82)	0.16	0.88
Diabetes	192	1.15	(0.99–1.35)	0.07	1.19	(1.02–1.39)	0.03	2.41	(0.91–6.37)	0.08	0.19
Respiratory Diseases	1,095	1.05	(0.98–1.12)	0.14	0.96	(0.90–1.02)	0.14	1.35	(0.90–2.02)	0.14	0.09
Cancer	3,687	1.03	(0.99–1.06)	0.16	1.00	(0.97–1.04)	0.78	1.18	(0.94–1.48)	0.16	0.15
Lung Cancer	612	1.04	(0.95–1.14)	0.43	0.95	(0.87–1.02)	0.17	1.27	(0.71–2.27)	0.43	0.31
Colorectal Cancer	543	1.03	(0.94–1.14)	0.49	0.99	(0.91–1.08)	0.82	1.23	(0.68–2.22)	0.49	0.46
Pancreatic Cancer	210	1.08	(0.93–1.26)	0.30	1.03	(0.89–1.19)	0.70	1.64	(0.65–4.13)	0.30	0.33

Stomach Cancer	193	0.89	(0.76–1.04)	0.15	0.92	(0.80–1.05)	0.20	0.48	(0.18–1.30)	0.15	0.21
External Causes	553	1.08	(0.99–1.18)	0.09	1.08	(0.99–1.18)	0.08	1.61	(0.92–2.82)	0.09	0.15

BMI: body mass index, CI: confidence interval, DBP: diastolic blood pressure, HR: hazard ratio, IV: instrumental variable, SD: standard deviation

adjusted for age, sex and HUNT survey of offspring/parent from whom DBP was measured, offspring and parental smoking status, age, DOB, alcohol consumption, education, own and spouse's employment, exercise and sex of parent

Supplementary Table S17. International classification of diseases (ICD) codes included for each cause of death

Cause of death	ICD 8 (1984-1985)	ICD 9 (1986-1995)	ICD 10 (1996-2009)
All cause	all	all	all
Cardiovascular disease[†]	3900-4441; 4444-4589; 7820-7829	3900-4599	I*
Coronary heart disease[‡]	4100-4149	4100-4149	I200-I259; I516
Stroke[§]	2930-2931; 4300-4389; 3440-3449	2904; 3420-3429; 3440-3449; 4300-4389	I600-I699; G450-G459
Diabetes[//]	2500-2509	2500-2509	E100-E149
Respiratory diseases[#]	4600-5199	4600-5199	J*
Cancer**	1400-2099	1400-2089; 2384; 2898	C*
Lung cancer	1620-1639	1620-1639	C330-C349
Breast cancer	1740-1749	1740-1759	C500-C509
Prostate cancer	1850-1859	1850-1859	C610-C619
Colorectal cancer	1530-1549	1530-1549	C180-C219
Pancreatic cancer	1570-1579	1570-1579	C250-C259
Stomach cancer	1510-1519	1510-1519	C160-C169
Ovarian cancer	1830	1830	C560-C569
External causes[††]	8000-9999	8000-9999	V*; W*; X*; Y*

ICD: international classification of diseases

**Any code beginning with the indicated letter*

[†]Cardiovascular disease consisted of all disease of the circulatory system listed in ICD 9 and 10, including coronary heart disease and stroke. Some conditions were either added or removed from the cardiovascular grouping in ICD 8 for consistency with ICD 9 and 10 (for example, 7820-7829 “Symptoms referable to cardiovascular and lymphatic system” was added to ICD 8).

[‡]Coronary heart disease is a narrowing of the arteries supplying the heart muscle and may be considered synonymous with ischemic heart disease or coronary artery disease.

[§]Stroke included bleeding from (haemorrhagic stroke) or blockage of (ischemic stroke) the arteries supplying the brain, as well as transient ischemic attacks (“mini-strokes”).

[||]Diabetes included insulin-dependent and non-insulin-dependent diabetes mellitus.

[#]Respiratory diseases included all non-neoplasmic diseases of the lungs, pleura and respiratory tract.

**Cancers excluded benign or in-situ neoplasms.

[††]External causes consisted of accidents and violence, including suicide and conditions consequent to accidents and violence.

Supplementary Table S18. P-values for parental comparison for adjusted observational HRs per SD of offspring and own SBP

Cause of Death	Deaths	P-value for Parental Comparison*	
		Offspring SBP, fully adjusted†	Own SBP, fully adjusted†
All cause	18,540	0.12	0.52
Cardiovascular Disease	8,772	0.06	0.62
Coronary Heart Disease	6,571	0.01	0.40
Stroke	2,268	0.69	0.31
Diabetes	312	0.73	0.71
Respiratory Diseases	1,418	0.18	0.28
Cancer	4,566	0.88	0.26
Lung Cancer	695	0.97	0.33
Colorectal Cancer	704	0.19	0.99
Pancreatic Cancer	273	0.12	0.24
Stomach Cancer	257	0.42	0.77
External Causes	691	0.95	0.94

HR: hazard ratio, SBP: systolic blood pressure, SD: standard deviation

**Z-test of an interaction between parental sex and the exposure, added to the standard model*

†adjusted for age, sex and HUNT survey of offspring/parent from whom SBP was measured, offspring and parental smoking status, age, DOB, alcohol consumption, education, own and spouse's employment, exercise and sex of parent

Supplementary Table S19. P-values for parental comparison for adjusted observational HRs per SD of offspring DBP

Cause of Death	Deaths	P-value for Parental Comparison*	
		Offspring DBP, fully adjusted†	Own DBP, fully adjusted†
All cause	18,540	0.05	0.53
Cardiovascular Disease	8,772	0.01	0.65
Coronary Heart Disease	6,571	0.03	0.88
Stroke	2,268	0.65	0.06
Diabetes	312	0.75	0.33
Respiratory Diseases	1,418	0.19	0.66
Cancer	4,566	0.62	0.55
Lung Cancer	695	0.89	0.07
Colorectal Cancer	704	0.59	0.79
Pancreatic Cancer	273	0.22	0.52
Stomach Cancer	257	0.61	0.60
External Causes	691	0.84	0.54

DBP: diastolic blood pressure, HR: hazard ratio, SD: standard deviation

**Z-test of an interaction between parental sex and the exposure, added to the standard model*

†adjusted for age, sex and HUNT survey of offspring/parent from whom DBP was measured, offspring and parental smoking status, age, DOB, alcohol consumption, education, own and spouse's employment, exercise and sex of parent

SUPPLEMENTARY FIGURES

Supplementary Figure S1: Flow chart of HUNT and Young-HUNT participants by mother-offspring/father-offspring pairs.

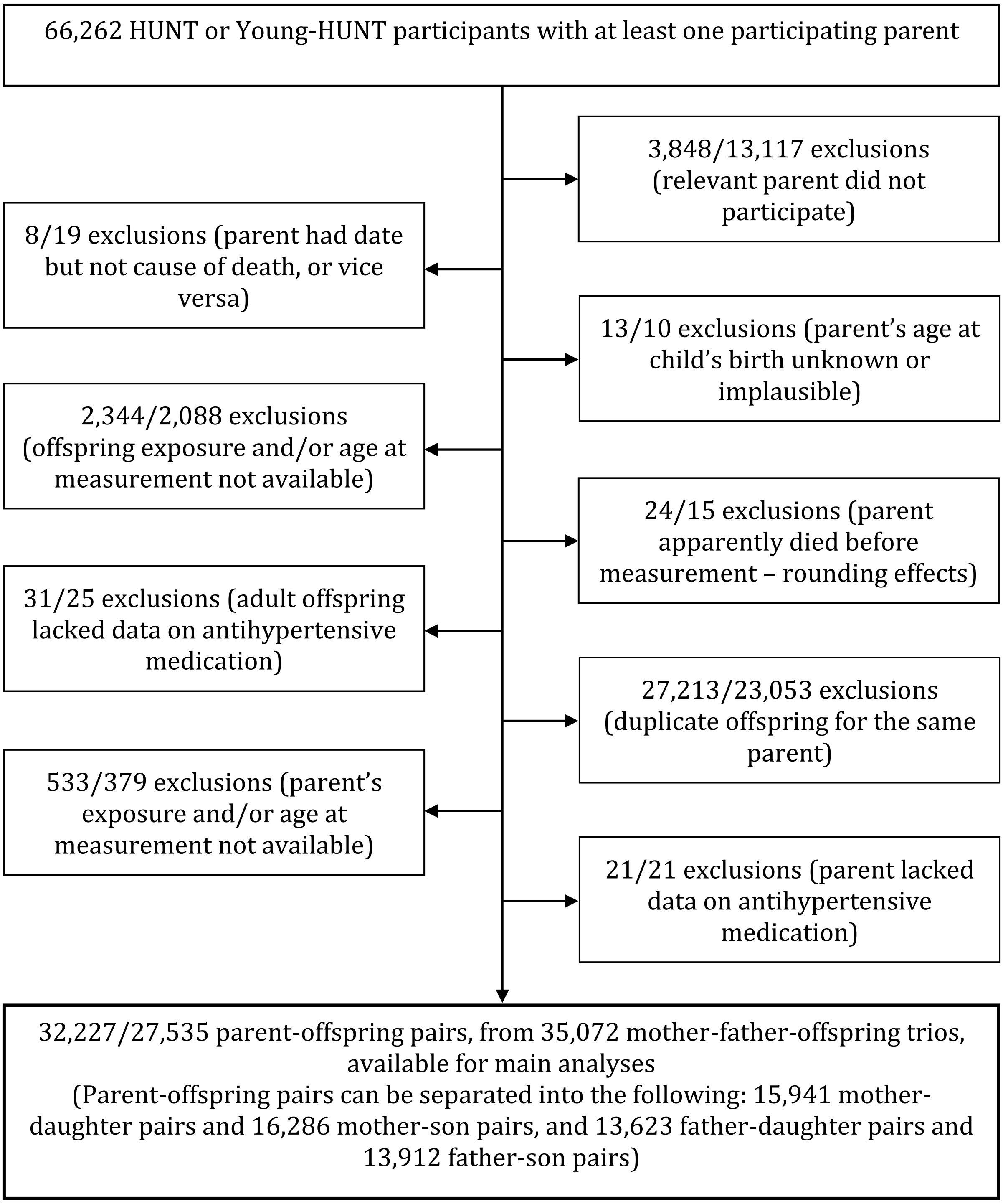

Supplementary Figure S2: Directed acyclic graph (DAG) of the underlying IV methodology employed in analyses and key assumptions for instrument validity. Offspring BP (BP_{off}) is a valid instrument for own BP (BP_{own}) providing that (1) BP_{off} is associated with BP_{own} (here, BP_{off} does not strictly cause BP_{own}, but is associated through common genetic and environmental factors (G/E)); (2) there is no independent association between BP_{off} and the outcome of interest (O); and (3) BP_{off} is not associated with any unmeasured confounding factors (C).

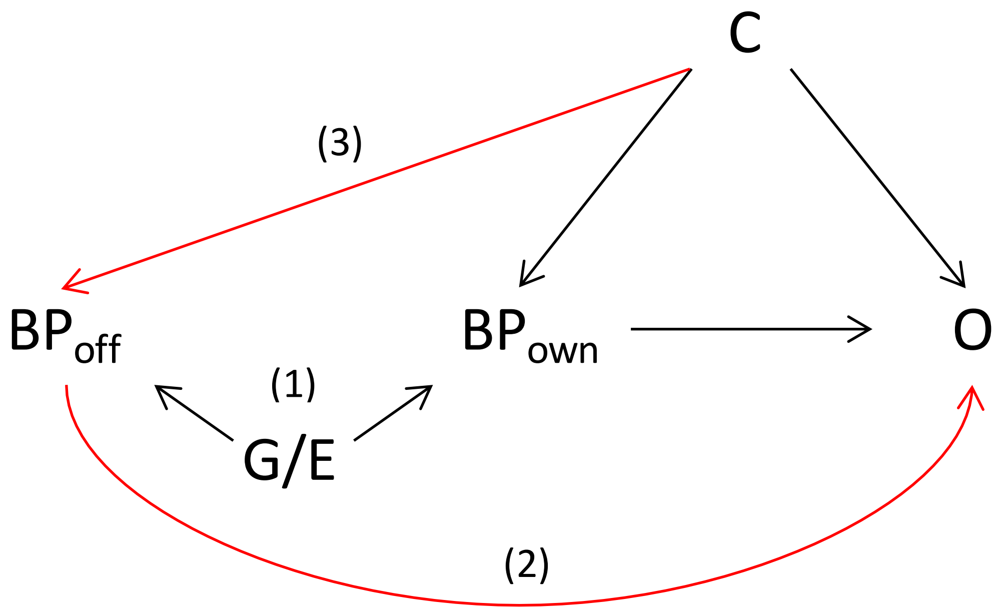

Supplementary Figure S3: Fitted HRs (median SBP as a reference) from cubic spline models of all-cause mortality, and mortality from cardiovascular disease, coronary heart disease, stroke, and diabetes against SBP. Shaded areas represent 95% CI and vertical lines represent the 1st and 99th percentiles of SBP. The upper X-axis applies to male SBP and the lower X-axis to female SBP.

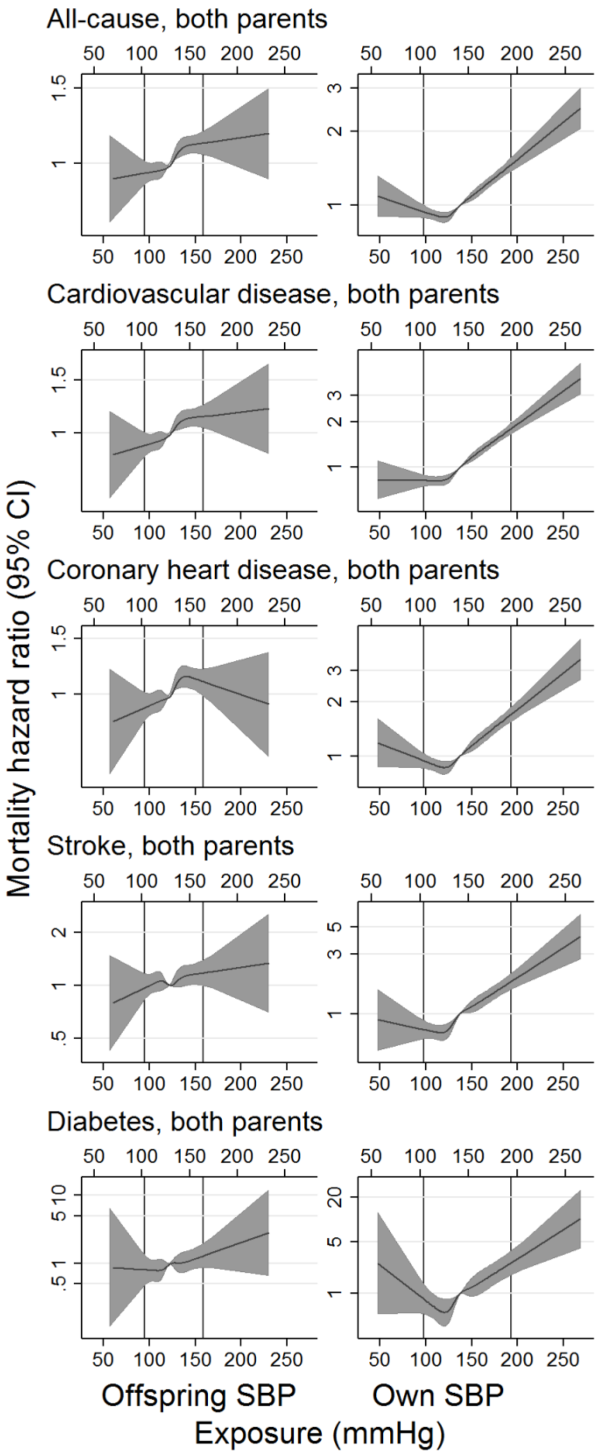

Supplementary Figure S4: Fitted HRs (median DBP as a reference) from cubic spline models of all-cause mortality, and mortality from cardiovascular disease, coronary heart disease, stroke, and diabetes against DBP. Shaded areas represent 95% CI and vertical lines represent the 1st and 99th percentiles of DBP. The upper X-axis applies to male DBP and the lower X-axis to female DBP.

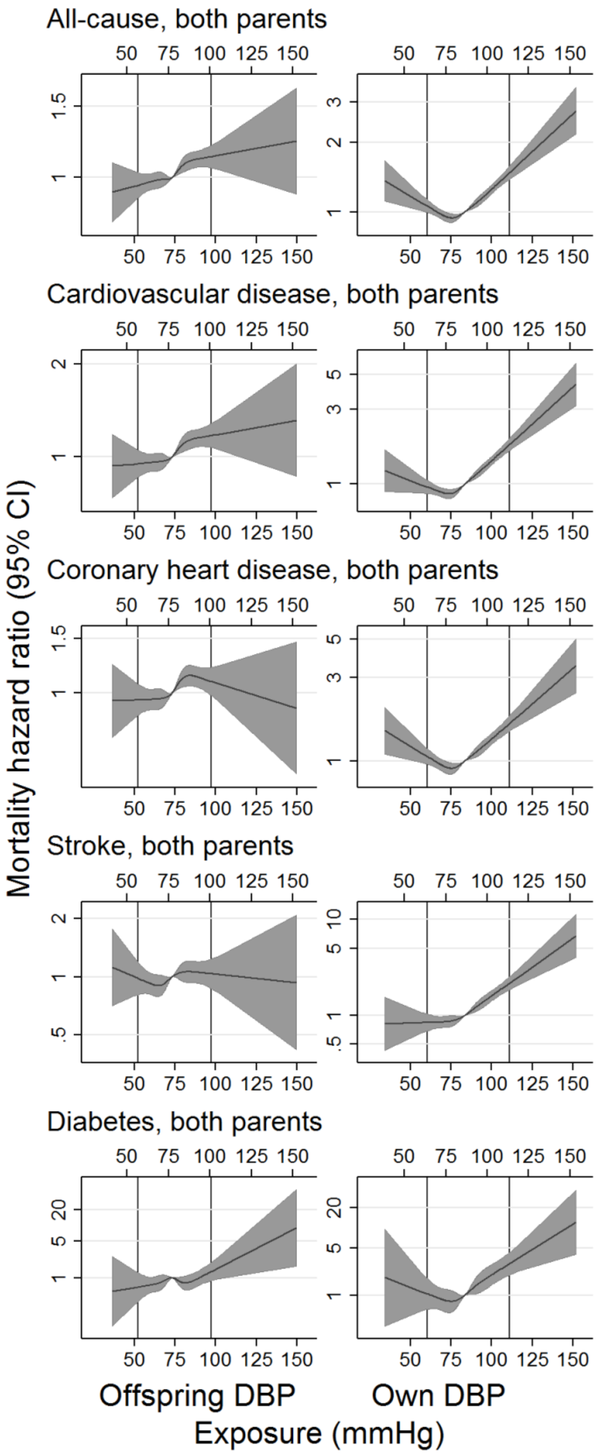

Supplementary Figure S5: Fitted HRs (median SBP as a reference) from cubic spline models of mortality from respiratory disease, cancer, lung cancer, breast cancer and prostate cancer against SBP. Shaded areas represent 95% CI and vertical lines represent the 1st and 99th percentiles of SBP. The upper X-axis applies to male SBP and the lower X-axis to female SBP.

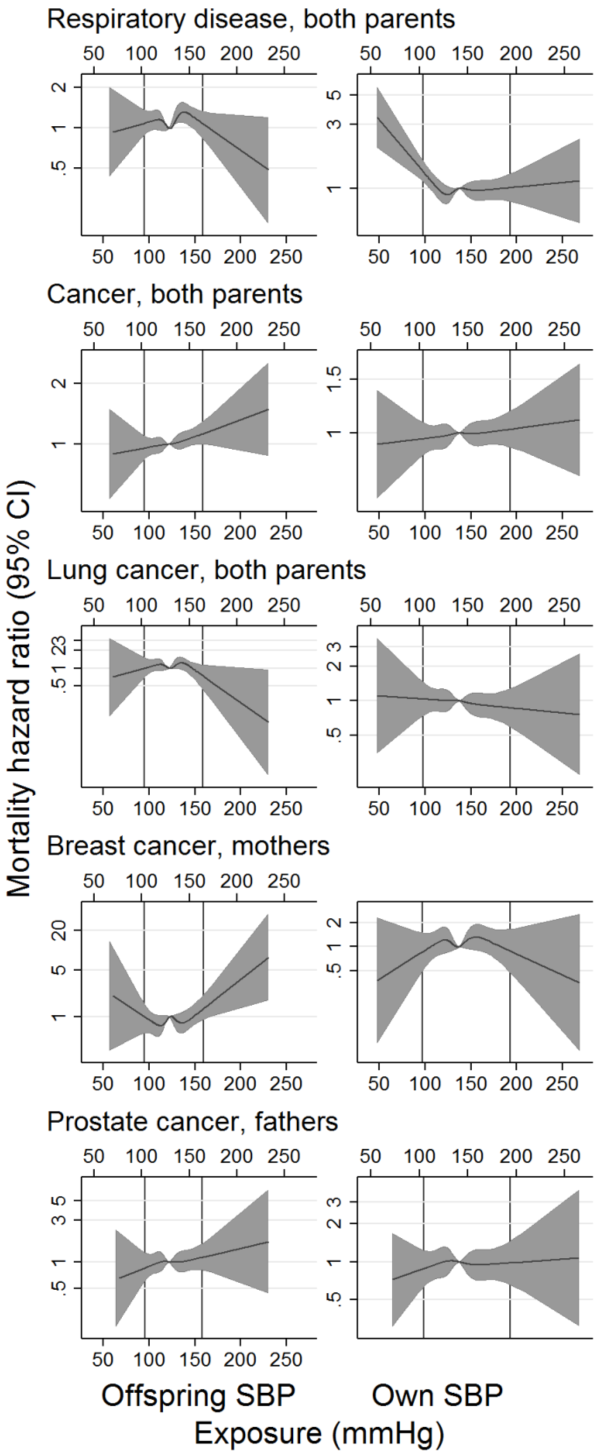

Supplementary Figure S6: Fitted HRs (median DBP as a reference) from cubic spline models of mortality from respiratory disease, cancer, lung cancer, breast cancer and prostate cancer against DBP. Shaded areas represent 95% CI and vertical lines represent the 1st and 99th percentiles of DBP. The upper X-axis applies to male DBP and the lower X-axis to female DBP.

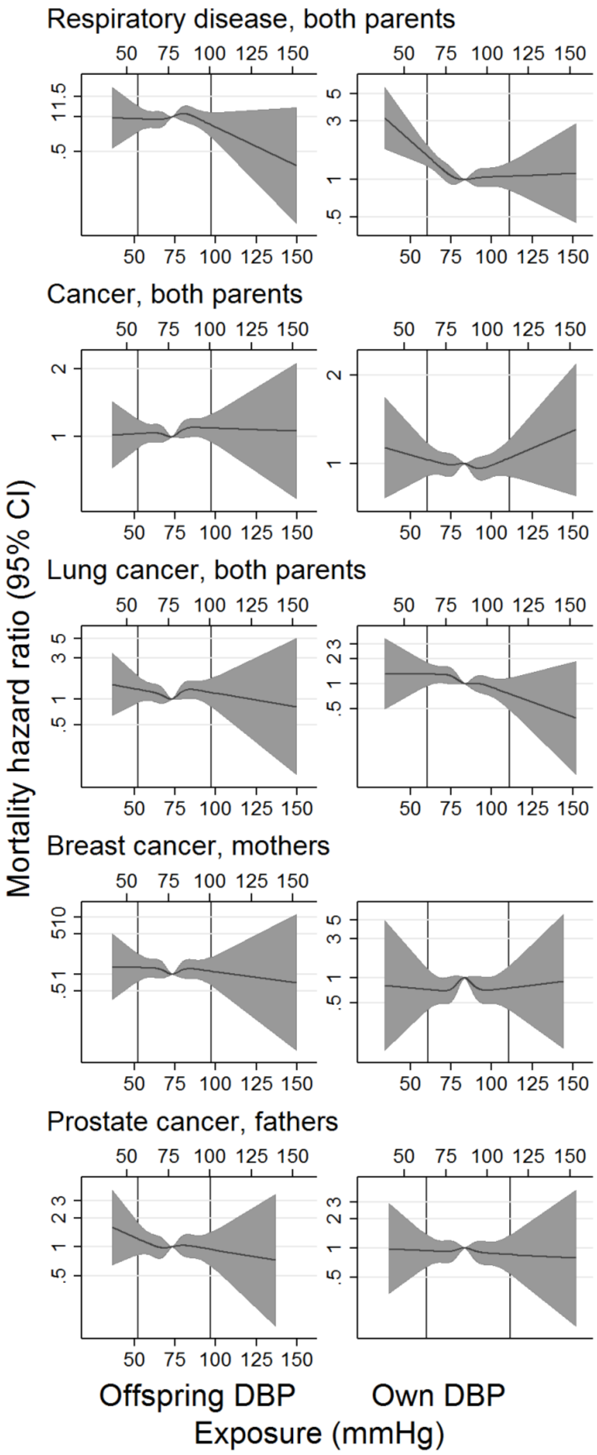

Supplementary Figure S7: Fitted HRs (median SBP as a reference) from cubic spline models of mortality from colorectal cancer, pancreatic cancer, stomach cancer, ovarian cancer and external causes against SBP. Shaded areas represent 95% CI and vertical lines represent the 1st and 99th percentiles of SBP. The upper X-axis applies to male SBP and the lower X-axis to female SBP.

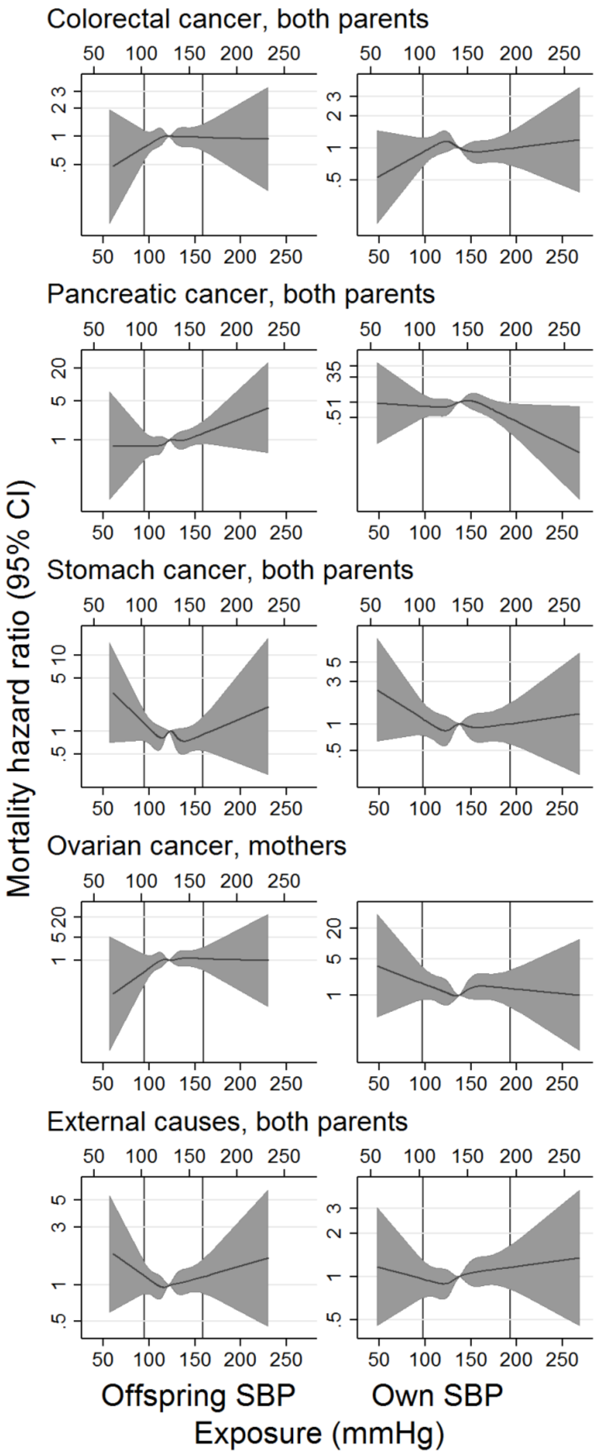

Supplementary Figure S8: Fitted HRs (median DBP as a reference) from cubic spline models of mortality from colorectal cancer, pancreatic cancer, stomach cancer, ovarian cancer and external causes against DBP. Shaded areas represent 95% CI and vertical lines represent the 1st and 99th percentiles of DBP. The upper X-axis applies to male BP and the lower X-axis to female DBP

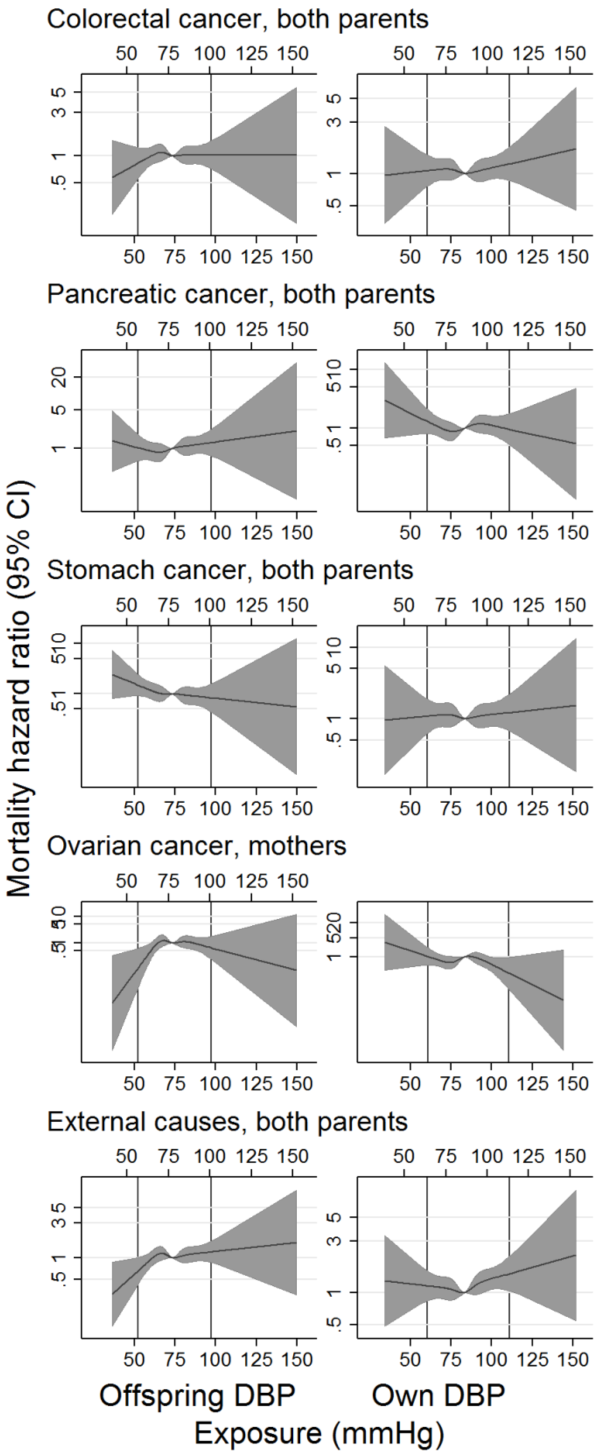

SUPPLEMENTARY REFERENCES

1. Thomas, D.C., Lawlor, D., Thompson, J.R.. Re: Estimation of bias in nongenetic observational studies using "Mendelian triangulation" by Bautista et al. *Ann Epidemiol.* **17,** 511-3 (2007).
